# The interaction of disease transmission, mortality, and economic output over the first 2 years of the COVID-19 pandemic

**DOI:** 10.1371/journal.pone.0301785

**Published:** 2024-06-13

**Authors:** Christian Morgenstern, Daniel J. Laydon, Charles Whittaker, Swapnil Mishra, David Haw, Samir Bhatt, Neil M. Ferguson

**Affiliations:** 1 MRC Centre for Global Infectious Disease Analysis & WHO Collaborating Centre for Infectious Disease Modelling, Jameel Institute, School of Public Health, Imperial College London, London, United Kingdom; 2 University of Copenhagen, Copenhagen, Denmark; Los Alamos National Laboratory, UNITED STATES

## Abstract

**Background:**

The COVID-19 pandemic has caused over 7.02 million deaths as of January 2024 and profoundly affected most countries’ Gross Domestic Product (GDP). Here, we study the interaction of SARS-CoV-2 transmission, mortality, and economic output between January 2020 and December 2022 across 25 European countries.

**Methods:**

We use a Bayesian mixed effects model with auto-regressive terms to estimate the temporal relationships between disease transmission, excess deaths, changes in economic output, transit mobility and non-pharmaceutical interventions (NPIs) across countries.

**Results:**

Disease transmission intensity (*logR*_*t*_) decreases GDP and increases excess deaths, where the latter association is longer-lasting. Changes in GDP as well as prior week transmission intensity are both negatively associated with each other (-0.241, 95% CrI: -0.295 - -0.189). We find evidence of risk-averse behaviour, as changes in transit and prior week transmission intensity are negatively associated (-0.055, 95% CrI: -0.074 to -0.036). Our results highlight a complex cost-benefit trade-off from individual NPIs. For example, banning international travel is associated with both increases in GDP (0.014, 0.002—0.025) and decreases in excess deaths (-0.014, 95% CrI: -0.028 - -0.001). Country-specific random effects, such as the poverty rate, are positively associated with excess deaths while the UN government effectiveness index is negatively associated with excess deaths.

**Interpretation:**

The interplay between transmission intensity, excess deaths, population mobility and economic output is highly complex, and none of these factors can be considered in isolation. Our results reinforce the intuitive idea that significant economic activity arises from diverse person-to-person interactions. Our analysis quantifies and highlights that the impact of disease on a given country is complex and multifaceted. Long-term economic impairments are not fully captured by our model, as well as long-term disease effects (Long COVID).

## Introduction

The World Health Organisation (WHO) declared a Public Health Emergency of International Concern (PHEIC) on 30 January 2020 as rising SARS-CoV-2 infections were detected across several countries. During the pandemic’s initial phase, governments enacted large-scale, and unprecedented non-pharmaceutical interventions (NPIs) to control disease transmission that had substantial impacts on society [1]. Countries introduced health containment measures and economic support interventions at different times and with different degrees of stringency to each other [2]. This in effect set up a longitudinal natural experiment [3], allowing us to assess the effectiveness of NPIs on the economy.

The worldwide economic impact of the COVID-19 pandemic has been severe [4]. However, most countries have since recovered their previous GDP. Countries offered significant support to their populations, particularly through furlough payments and business continuity support [2]. Sole economic costs of the pandemic do not account for other wide-ranging societal impacts. Prolonged school closures led to loss of learning for children, which will likely lead to long-term outcomes, and may more generally result in a loss of national GDP over the coming decades [5, 6].

Between 4 January 2020 and 21 January 2024 (the WHO announced on 5 May 2023 the end of the PHEIC [7]), there were 774 million confirmed cases and over 7 million confirmed deaths [8]. These official counts underestimate true COVID-19 burden to varying extents between countries [9, 10]. More positively, the COVID-19 pandemic also saw rapid vaccine development and rollout [11]. The UK was the first country to administer a COVID-19 vaccine, beginning on 8 December 2020. By the end of the PHEIC, over 13.38 billion doses of COVID-19 vaccines have been administered, enabling societies to gradually return to normal life and significantly lessen the health and economic burden of COVID-19 [12].

The COVID-19 pandemic has generated unparalleled quantities of data, much of which is publicly available. Using these extensive and granular data sets, which we review in the Methods section, we explore the interaction between transmission intensities (*log* of the real-time reproduction number *R*_*t*_), excess deaths, mobility and economic output. We use previously published estimates of the real-time reproduction number as well as *GDP* and consider their interaction with NPIs using a Bayesian mixed effects model with auto-regressive terms.

Much early research focused on estimating the impact of NPIs on disease transmission. The heterogeneity in NPI implementation and stringency, both over time and by location, has been used to previously assess the association between disease transmission and NPIs. Mechanistic models using case data are commonly used to estimate time-varying reproductive numbers [13–15], especially during the early phases of a pandemic. We use previously published, and widely used, estimates of the real-time reproduction number from [15] in our work. Semi-mechanistic models of *R*_*t*_, such as Bhatt et al [16], are used to infer *latent* transmission rates. The use of multiple data streams (such as cases, deaths) improves robustness and allows concurrent estimation of NPIs’ effectiveness and the time-varying reproduction number *R*_*t*_. This integrated approach involves parameterising *R*_*t*_ using multi-level Bayesian regressions, with NPIs as covariates. Flaxman et al [17] considered binary variables for NPIs across 11 European countries and found that only ‘lockdown’ had an identifiable and substantial impact on reducing transmission. Brauner et al [18] implemented a similar model, using a curated set of NPIs across a wider set of countries, to obtain improved estimates of effect sizes. Importantly Brauner et al and Liu et al reach similar conclusions regarding NPI impact using different methods, strengthening their conclusions.

There has been far less work investigating the impact of NPIs on economic activity. Turner et al [19] implement a model similar to Liu et al [20], but with the percentage of weekly GDP versus pre-pandemic GDP as a counterfactual response variable. Their longitudinal model considers 33 countries between January 2020 and May 2021. They found that NPIs for health reduces GDP relative to the pre-pandemic counterfactual. Adda [21] explored a similar type of longitudinal regression model investigating the association of epidemiological variables and economic variables for flu-like illnesses, acute diarrhoea and chickenpox.

There has further been a significant focus on mobility and its association with disease transmission. Unwin et al [22, 23] used mobility to capture the impact of NPIs and behaviour more generally for the United States at the state level. The advantage of mobility is that it captures what individuals actually do, and not merely the restrictions (NPIs) they are subjected to, where the latter may not be adhered to. The disadvantage of mobility data is that it is usually analysed as an aggregated measure that does not allow a more granular policy analysis.

Individual behaviour has long been recognized as a crucial determinant of epidemic trajectory. However, there is an absence of data that can reliably inform theoretical models, which have therefore focused on the trade-offs of human-to-human contact given transmission dynamics. Fenichel et al [24] showed that behaviour should be included in infectious disease modelling, as it impacts parameter estimation and therefore the design of social distancing interventions.

Studies and models considering multiple countries are relatively rare. Famiglietti et al [25] proposes a structural vector autoregression model considering deaths, health containment measures (using OxGRT stringency on C measures) and exports, as a measure of economic activity, as response variables, based on US state-level data. They find that NPIs are highly effective at reducing deaths and that they have no long-term impact on export activity. However, their analysis has several drawbacks (e.g. focusing on deaths and not excess deaths). Camehl et al [26] follow a similar approach considering deaths, mobility, and containment policy stringency.

There are several other variables which could be considered to gain better insights into the spatial diffusion dynamics of the COVID-19 pandemic. Bontempi [27] considers air pollution across US states as an indicator for the spread of COVID-19 and commercial trade. Similarly, Bontempi et al [28] considered if commercial trade could be an indicator of COVID-19 spread in Italy, Spain, and France. Qiu et al [29] considers environmental and economic factors to understand the spread of COVID-19 across China and Gu et al [30] considers a spatial model of disease spread across China.

In this paper, we perform a statistical, retrospective analysis of the interaction between epidemiological variables and economic output.

## Materials and methods

In this section, we describe the data used and outline our Bayesian mixed effects model.

We consider data on transmission dynamics using the real-time reproduction number. In our model we also consider non-pharmaceutical interventions across containment and closure policies. Economic policies, and health system policies, and economic output measured by GDP nowcasts. We use mobility as a proxy for behaviour and excess mortality for 25 European countries from Jan 2020 to Dec 2021. Countries implemented interventions at different times and to different levels of stringency. The heterogeneity in interventions, in both space and time, sets up a natural experiment to study their effectiveness [3].

**Fig 1:**
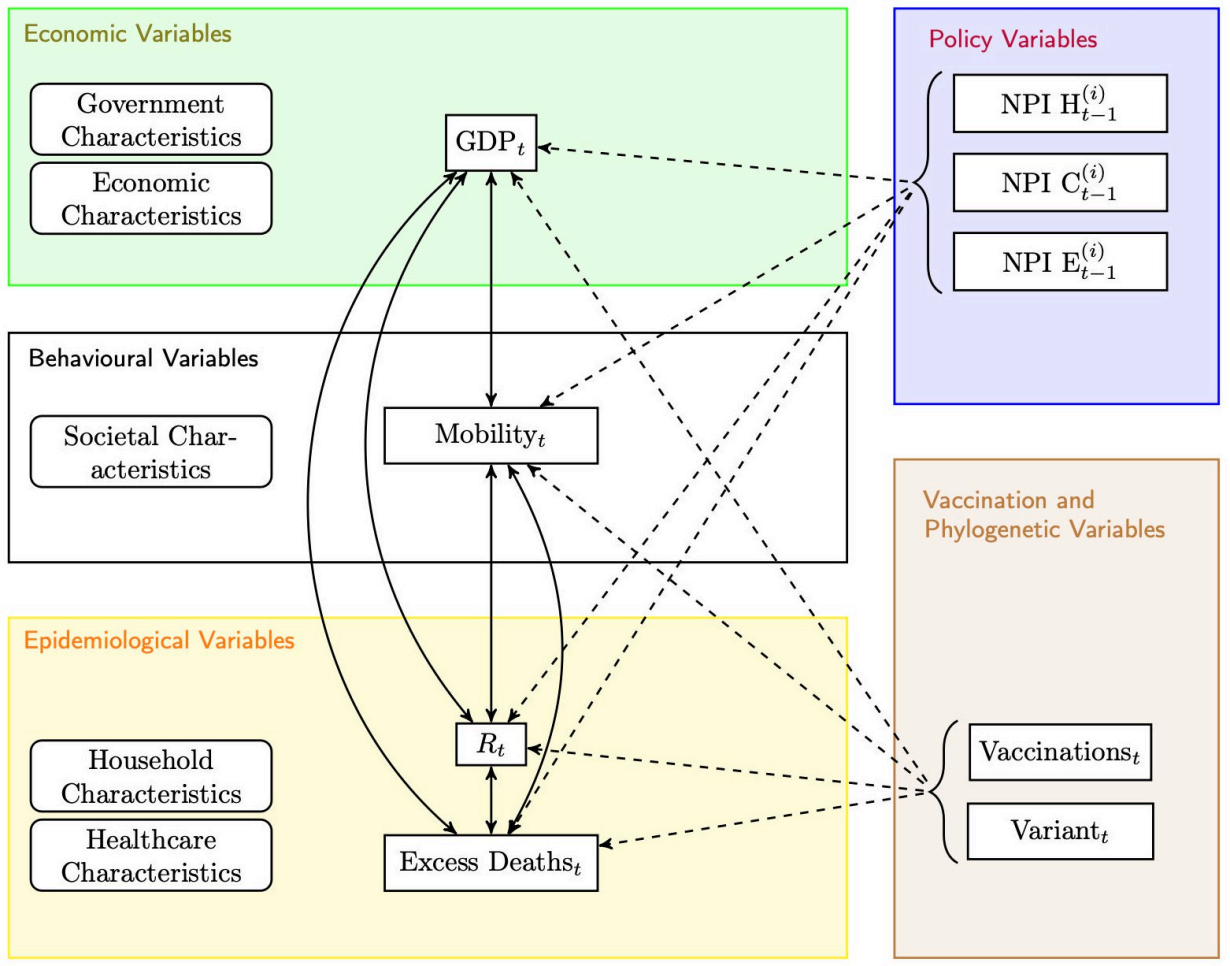
Schematic model representation. Interaction of economic (top box), behavioural (middle box) and epidemiological variables (bottom box) are represented on the left-hand side. Policy variables and variables related to vaccination and phylogenetics (right-hand side of schema) are explanatory variables only. NPI H, C, and E are non-pharmaceutical interventions for containment and closure policies, health system policies, and economic policies respectively.

Fig 1 provides a schematic representation of the variables considered.

### Data

#### Reproduction numbers *R*_*t*_

We use existing estimates of the real-time reproduction number *R*_*t*_ from the package EpiNow2 [15]. EpiNow2 is based on reported cases and calculated over a 12-week window, and has been validated against several other models [31, 32]. It is based on aggregated data which has the advantage of providing a consistent estimation of *R*_*t*_ across countries as the same methodology is applied. The *R*_*t*_ estimates may be affected by different case definitions and different levels of testing or reporting, which may impact the ascertainment ratio, i.e. the proportion of infections identified, to varying degrees between different countries. We provide a plot of *logR*_*t*_ in Figure A.1 of the S1 File. In the model, the daily median of the *R*_*t*_ estimates for a country is used and the data was retrieved from [33].

#### NPI data

NPI data is obtained from the Oxford Blavatnik School of Government Covid-19 Government Response Tracker (OxCGRT) [2], retrieved from [34]. Daily data is available by country, intervention, and strength of intervention (scale dependent on type of intervention). Interventions are grouped into containment and closure policies, economic policies and health system policies. A full list of interventions, including descriptions and scales, are available in Tables A.1–A.3 and a plot of NPIs in Figure A.6 of the S1 File.

#### Economic data

Economic data is typically only reported on a monthly or quarterly basis, presenting a challenge when studying the interaction between the economy and the pandemic. Before the pandemic, the OECD proposed a nowcasting methodology for modelling weekly GDPs which we accessed via GitHub [35] and we are using weekly index levels of the OECD Tracker by country. The OECD created a two-step model using 215 categories and 33 topics related to economic activity from a panel of Google Trends data. To assess economic impact, we consider changes in economic activity as measured by the weekly GDP index and economic activity, adjusted for pre-pandemic growth trends (mean growth rate between 2016 and 2019 for each country). Removing the pre-pandemic growth trend adjusts for inherent differences between the economies of different countries. A plot of indexed GDP over the study period is included in Figure A.4 in the S1 File.

#### Excess mortality data

Countries have varying definitions of what constitutes a Covid-19 death. Testing capacity varied widely, especially in the initial phase of the pandemic. Therefore lab-confirmed deaths underestimate true Covid-19 deaths. To correct for this underestimate, we use estimated excess deaths [36] and retrieve the data from github (TheEconomist/covid-19-the-economist-global-excess-deaths-model) [37] (Figure A.2 in S1 File), which is consistent with work by WHO [38]. We note that there has been much discussion on excess deaths in the literature (see e.g. Bager et al [39]). The data is provided weekly as excess deaths per 100k population for each country. We also account for the delay between infection and death, using the mean symptom onset-to-death delay of 15 days [40]).

#### Mobility data

Mobility data is of significant interest as it captures the actual behaviour of individuals. Google provided mobility data over the pandemic period across a wide range of countries, as well as categories for which mobility could be recorded: retail and recreation, groceries and pharmacies, parks, transit stations, workplaces, and residential. We retrieved the data from [41] and focus on transit stations, as these represent mobility outside the home that involves contacts with non-household members, by the nature of mass transit relative to travelling by car, and they are a proxy for risk-seeking behaviour, either to capture economic opportunities or for non-essential leisure activities. This data is available at the country level daily as an index. The index is presented as the percentage change from mobility in each category, where the baseline is the median value for each weekday, between 3 Jan and 6 Feb 2020 (Figure A.3 in S1 File).

#### Variant data

We obtained SARS-CoV-2 variant data from [42] for each country and week (based on GISAID data) and retrieved the data from github (*hodcroftlab*/*covariants*/*master*/*cluster*_*tables*) [43]. The data is provided at the nextstrain clade level, which we aggregate into WHO labels (e.g. Alpha). We choose the majority strain as the dominant strain for each week and country. We estimate missing data for Hungary, weeks 9–45 of 2021 by taking the weighted average of neighbouring countries, with weights equal to the percentage of border length shared with that country, choosing the dominant strain by majority vote. A plot of the dominant variant by country is provided in Figure A.9 in the S1 File.

#### Vaccination data

We use the average number of vaccinations per person. The total number of vaccinations administered is obtained from Our World In Data [8] and retrieved from [44], (Figure A.8 in S1 File). More granular data, disaggregated by age group, would offer an improvement, but we were not able to find consistent data across the countries of interest.

#### Country characteristics data

It is important to consider a range of societal, economic, governance and health systems data when studying the interaction of the economy and the pandemic. Each of these vary over time, but not fast enough to be relevant to our analysis given the time-frame we consider. We provide an overview of these characteristics in Table A.4 in the S1 File and consider these as constants for our analysis.

#### Model

To investigate the interaction of SARS-CoV-2 transmission, mortality and the economy, we use a Bayesian mixed effects model with auto-regressive terms, which we fit using stan (via brms) [45, 46]. In this section we present our main model. We provide detailed specification and results for two special cases of our main model in the (section B.2 in S1 File).

The model is a Bayesian hierarchical model and any reference to random and fixed effects should be considered in the context of that modelling framework.

#### Scaling of data

Because we use stan for parameter inference, we must ensure all variables are scaled to be centred around zero, and that the scale of all variables is approximately the same. This is important to obtain efficient sampling.

- We log transform the instantaneous reproduction number and use log *R*_*t*_ (transmission intensity).
- We consider the log of excess deaths per 100k.
- We consider changes in the overall stringency index divided by 100.
- We consider changes in GDP divided by 10.
- We consider changes in Transit divided by 100.

#### Model

*Y*_*t*,*c*_ is a multivariate normal random variable with covariance matrix Σ_*u*_. *y*_*t*,*c*_ is a vector containing the values of the response variables, as defined in Eq 2, at time *t* for country *c* which we model as a vector auto-regressive process of order *p* for *N* = 25 European countries. *μ*_*c*_ represent country-specific random effects. *Φ*_*k*_ is the *N* × *N* coefficient matrix of the vector auto-regressive component for lag *k*. *ν* is the coefficient for vaccination (defined as the average number of vaccinations per person at time *t* for country *c*). *ψ* is the coefficient for the *j*^*th*^ dominant variant of SARS-CoV-2. Ψ_*j*,*t*, *c*_ is constructed such that Ψ_*WT*,⋅,⋅_ is always 1 (and acts as an intercept term), Ψ_*Alpha*,⋅,⋅_ is 1 unless Wildtype is the dominant SARS-CoV-2 variant (in which case it is 0), Ψ_*Delta*,⋅,⋅_ is 1 unless Wildtype or Alpha are the dominant variants and Ψ_*Omicron*,⋅,⋅_ is 1 only if Omicron is the dominant variant. Stringency of individual NPIs (across containment and closures, economic and health system policies) are represented by the vector *x*_*t*,*c*_ and changes in stringency are represented as Δ*x*_*t*,*c*_.

We consider individual NPIs to be covariates to the response variables (transmission intensity, excess deaths, changes in economic output and transit mobility). The implementation of NPIs, both in timing and stringency, have varied between countries. Early in the pandemic (March to September 2020), implementations of NPIs were reasonably homogeneous, but we subsequently observe significant heterogeneity, both over time and between countries. In this model, we consider *lagged* NPIs, where the coefficients λ and *δ* respectively denote the coefficients for stringency and changes in NPIs.

$$\begin{array}{ccc} Y_{t,c} & ∼ & \text{MVN}\left(y_{t,c},\Sigma_{u}\right)\\ y_{t,c} & = & \mu_{c}+\overset{p}{\underset{k=1}{\sum }}\Phi_{k}y_{t-k,c}+\lambda \cdot x_{t,c}+\delta \cdot \Delta x_{t,c}+\\ \; & \; & \nu \cdot vacc_{t,c}+\psi \cdot \sum_{\begin{array}{l} j\in \{WT,Alpha,\\ Delta,Omicron\} \end{array}}\Psi_{j,t,c} \end{array}$$ (1)

*where*:

$$\begin{array}{ccc} y_{t,c} & = & {\left(y_{1t,c},y_{2t,c},y_{3t,c},y_{4t,c}\right)}^{⊤}\\ & = & {\left({\text{log}R}_{t},\text{log}{\text{Excess}\;\text{Deaths}}_{t},\Delta {\text{GDP}}_{t},\Delta {\text{Transit}}_{t},\right)}^{⊤}\\ x_{t,c} & = & {\text{NPI}}_{t-1,c}\\ vacc_{t,c} & = & {\text{Average}\;\text{Vaccinations}\;\text{per}\;\text{person}}_{t} \end{array}$$ (2)

#### Common priors and parameters across models

We use normal priors with zero mean and variance *τ* on the response variables in the vector auto-regressive part of the model. We assume the same prior *N*(0, *τ*) for all other explanatory variable coefficients. We manually choose *τ* to be 1, to give an uninformative prior. For country specific intercepts, we take a *N*(0, *σ*^2^) prior, where *σ* has a *Cauchy*(0, 2) prior. At the group level, parameters u are assumed to be multivariate normal with unknown covariance Σ_*u*_, which is further decomposed into variances (*σ*_1_, ⋯, *σ*_*N*_) and a correlation matrix *Ω*_*N*_ which has a LKJ prior [46] with hyperparameter *ξ* = 2.

$$\begin{array}{ccc} \Phi_{k,i,j} & ∼ & N\left(0,\tau^{2}\right)\;\;\;\forall k=1,i\in \left[1,N\right],j\in \left[1,N\right]\\ \lambda_{i},\delta_{i},\nu_{i},\psi_{i} & ∼ & N(0,\tau^{2})\;\;\;\forall i\\ \mu_{c} & ∼ & N(0,\sigma^{2})\;\;\;\\ \sigma & ∼ & cauchy(0,2)\\ \Omega_{N} & ∼ & \text{LKJ}(\xi ) \end{array}$$

The chosen priors are uninformative, as we assume a zero mean for all coefficients and *η* = 2 > 1, favouring less correlation amongst our parameters. We perform a range of sensitivity analyses, which we provide in the S1 File with the parameter ranges provided in Table 1.

**Table 1:** Table of parameters used in modelling.

Parameter	Type	Value	Range	Description	Results
p	VAR	1		order of VAR process	-
c	VAR	Country	LOO*	country index	Fig C.20 in S1 File
N	VAR	25		number of countries	-
Σ_*u*_	VAR	fitted		covariance matrix	
*μ*_*c*_	VAR	fitted		random effects	Fig 4
Φ_*k*_	VAR	fitted		coefficients for response variables	Table C.5 in S1 File
λ	VAR	fitted		coefficients for stringency of NPIs	Table C.6 in S1 File
*δ*	VAR	fitted		coefficients for changes of NPIs	Table C.7 in S1 File
*ψ*	VAR	fitted		coefficients for dominant variant	Table C.8 in S1 File
*ν*	VAR	fitted		coefficients for vaccination	Table C.9 in S1 File
*τ*	prior	1	(0.2,2)	prior on response variable coefficients	-
*σ*_*i*_	prior	cauchy(0,2)		prior on country random effects	-
*ξ*	prior	2	(1,2,4)	hyperparameter on LKJ prior	-
adapt_delta	Stan	0.9		target average acceptance probability	-
step_size	Stan	0.01		discretization interval	-
max_treedepth	Stan	10		max binary tree size in the NUTS algorithm	-

(*) LOO—leave one out sensitivity analysis

#### Identification strategy

We use Orthogonal Impulse Response Functions to assess the temporal relationships between variables. The Impulse Response Functions quantify the impact of a unit shock in one variable on all the remaining variables [47, 48]. The model we consider is over-identified, as we cannot deduce the impact of contemporaneous shocks on each variable. The model therefore requires constraints. This over-identification arises as our covariance matrix can be represented as $\Sigma_{u}=E\left[u_{t}^{⊤}u_{t}\right]=BB^{⊤}$, where *u*_*t*_ are the error terms of the VAR model and *B* is a matrix representing the shocks as a linear combination of our error terms (for an example see section B.3 in the S1 File). Here we constrain the contemporaneous impact of variables at time *t*, i.e. variable *A* does not immediately impact variable *B* (also referred to as a ‘short run restriction’). This is implemented by choosing *B* = *L* such that Σ_*u*_ = *LL*^⊤^ and *L* is a lower triangular matrix (that is *L* is the lower triangular Cholesky decomposition of Σ_*u*_). This resolves the identification problem for shocks. Below we justify these restrictions. Note that the ordering of the response variables matters in this identification scheme.

1. Transmission intensity cannot be affected contemporaneously by any other variable. Individuals change their behaviour subject to *past* information, which may lead to risk-seeking behaviour, such as searching for economic activity, or risk-mitigating behaviour, such as staying at home to avoid person-to-person contact.
2. Excess Deaths are affected by disease transmission (with a lag derived from the onset-to-death distribution of SARS-CoV-2) but are not immediately affected by any other variable. As with transmission, person-to-person contact is calibrated on past information.
3. NPIs are affected by disease transmissions and excess deaths (assuming that policymaking is instantaneous and translates observed disease dynamics into policy). However, individuals’ behaviours, such as mobility or economic activity, are not known to policymakers contemporaneously and hence do not affect NPIs contemporaneously.
4. Economic activity is not contemporaneously impacted by mobility since individuals’ observation of past economic activity drives their behaviour. Economic opportunity. as observed in the preceding time periods, drives transit behaviour and hence cannot impact Economic activity.
5. Transit mobility has no restrictions and can be affected by any other variable.

Other identification strategies are possible, and include ‘zero long-run effects’ and ‘sign restrictions’. We prefer the short-run restrictions set out above as they naturally fit with the disease transmission mechanism and human behaviour.

## Results

We provide results of the model described in this section. We provide results from a variation of the model in the S1 File as sensitivity analysis.

**Fig 2:**
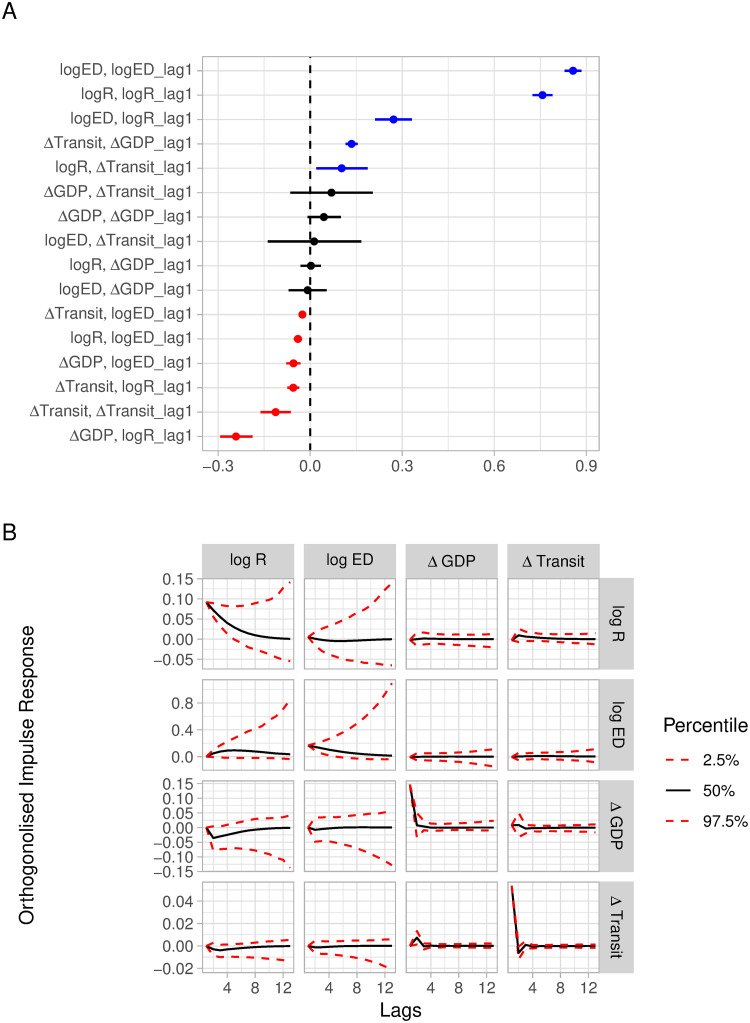
Model estimates. A. Vector auto-regressive coefficients and 95% credible intervals (CrI). Positive and significant coefficients are blue, negative and significant coefficients are red. B. Orthogonalised IRF. Columns are the variables to which the shock is applied to, rows are the variables which we observe the impulse response for. Mean estimates (solid black) and 95% CrI (dotted red). (Vector auto-regressive coefficient estimates are available in Table C.5 in the S1 File).

We first consider the results of the Orthogonal Impulse Response Function (Fig 2). A positive impulse in transmission intensity leads to an increase in excess deaths, peaking after 5 weeks, and the decay of the impulse is slow and prolonged. We observe a significant and negative response in economic activity (change in GDP), but no significant impact on changes in transit behaviour. The effect of a positive impulse in excess deaths is smaller than that for transmission intensity on both transmission intensity and economic activity. A positive impulse in economic activity has a positive effect on itself and a significant positive effect on transit mobility in the next time period.

Vector auto-regressive coefficients are given in Fig 2(B) and correspond to the parameter estimates of Φ_*k*_. The auto-regressive terms of the response variables lagged with themselves are significant for transmission intensity (0.757, 95% CrI: 0.725 to 0.790) and excess deaths 0.856 (95% CrI: 0.828 to 0.884). For changes in transit mobility, we observe a negative auto-regressive coefficient -0.113 (95% CrI: -0.163 to -0.063) and the auto-regressive coefficient for economic activity is not significant.

We highlight the significant, off diagonal vector auto-regressive coefficients: excess deaths and lagged transmission intensity are positively associated (0.271, 95% CrI: 0.213–0.329), consistent with a positive correlation between excess deaths and transmission. Transmission intensity and lagged changes in transit are positively associated (0.103, 95% CrI: 0.019–0.187) consistent with person-to-person interactions in the previous time period leading to transmission events. Changes in transit mobility are positively associated with lagged changes in economic activity (0.135, 95% CrI: 0.115 to 0.156) indicating that individuals react to observed economic activity and opportunity.

Economic activity is negatively associated with lagged transmission intensity (-0.241, 95% CrI: -0.295 to -0.189) and changes in transit is negatively associated with lagged transmission intensity (-0.055, 95% CrI: -0.074 to -0.036) implying that high transmission intensity leads to lower economic activity and transit mobility.

Transmission intensity and excess deaths are negatively associated (-0.040, 95% CrI: -0.054 to -0.026) as well as changes in transit mobility and excess deaths (-0.025, 95% CrI: -0.033 to—0.018) indicating that observed excess deaths seems to lead to a small, but significant, reduction in transmission and transit behaviour.

NPIs were implemented to varying degrees across countries and over time. The model includes NPIs as covariates in both their stringency in each week and the change in stringency from the previous week, as this improved model performance. We used Leave-One-Out cross-validation (LOO-CV) to estimate the pointwise out-of-sample prediction accuracy of our fitted Bayesian model [49]. The model with both stringency and changes outperformed the model which includes only the stringency of NPIs, with an expected log pointwise predictive density difference (ELPD-diff) of -80.9 (95% CrI: -136.1 to -25.7). The model with both stringency and changes is not statistically different from the model, which includes only changes in stringency (ELPD-diff of -23.8 (95% CrI: -54.8—7.2)). However, we prefer the model with both stringency and changes as it allows us to explain the effect of NPIs more fully.

**Fig 3:**
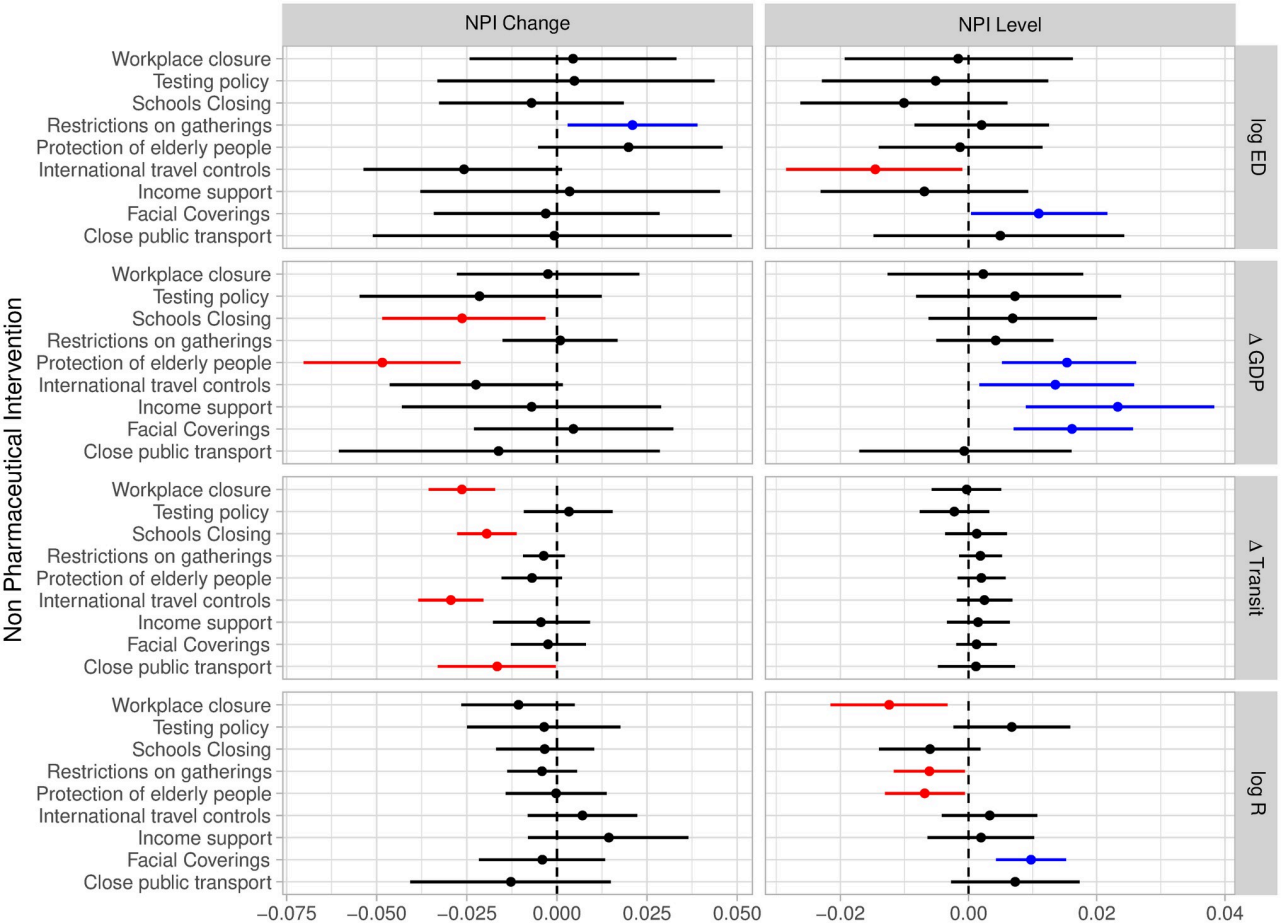
NPI effect sizes for model. Coefficient effect sizes (with 95% credible intervals) for each response variable. NPI changes (left column) and NPI stringency (right column) with NPI names listed on the vertical axis. Blue highlighted results indicate positive and significant coefficients and red indicates negative and significant coefficients.

Fig 3 shows the effect sizes for both the stringency of NPIs and changes therein. Excess deaths are positively associated with changes in restrictions on gatherings (0.021, 95% CrI: 0.003–0.039); negatively associated with the stringency of international travel controls (-0.014, 95% CrI: -0.028 to -0.001), and positively associated with the stringency of facial covering mandates (0.011, 95% CrI: 0.000–0.021). Economic activity is negatively associated with both changes in school closures (-0.026, 95% CrI: -0.049 to -0.003) and protection of the elderly (-0.049, 95% CrI: -0.071 to -0.027). Economic activity is positively associated with the stringency of protection of the elderly (0.015, 95% CrI: 0.005 to 0.027), international travel controls (0.014, 95% CrI: 0.002 to 0.025), income support (0.023, 95% CrI: 0.009 to 0.039) and facial coverings (0.016, 95% CrI: 0.007 to 0.026). Changes in transit behaviour were negatively associated with changes in mandate level for workplace closure (-0.026, 95% CrI: -0.036 to -0.017), school closure (-0.019, 95% CrI: -0.028 to -0.011), international travel controls (-0.030, 95% CrI: -0.038 to -0.021) and closure of public transport (-0.017, 95% CrI: -0.033 to -0). Transmission intensity was negatively associated with the mandate level for workplace closures (-0.012, 95% CrI: -0.021 to -0.003), restrictions on gatherings (-0.006, 95% CrI: -0.012 to -0.001), protection of the elderly (-0.007, 95% CrI: -0.013 to -0.000) but positively associated with the mandate level for facial coverings (0.01, 95% CrI: 0.004–0.015).

If only changes in NPIs or only stringency of NPIs are included in the model we obtain largely similar results, which are reported in the S1 File.

**Fig 4:**
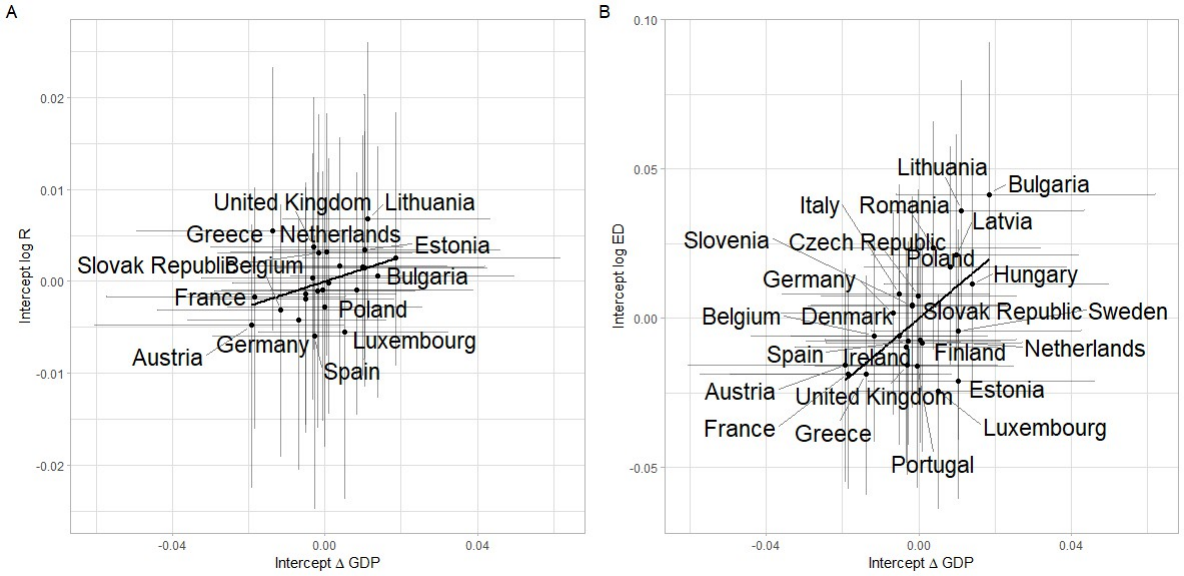
(A.) Country-specific intercepts *μ*_*c*_ for transmission intensity vs economic activity. (B.) Country-specific *μ*_*c*_ intercepts for excess deaths vs economic activity.

We consider the results for *μ*_*c*_, the country-specific effects (random effects) (Fig 4). We first note that there is very little unexplained variability in changes in transit mobility. Considering excess deaths and transmission intensities we observe that the order of countries is similar for both. This is perhaps unsurprising given the strong link between transmission and excess deaths, where differences are driven by the demography, health status and health systems of countries. Correlations for country intercepts between excess deaths and economic activity is 0.23 (95% CrI: -0.21 to 0.59) and similarly for transmission intensity and economic activity the correlation coefficient is 0.09 (95% CrI: -0.33 to 0.46), which suggests a trade-off between excess deaths and economic activity, irrespective of interventions.

**Fig 5:**
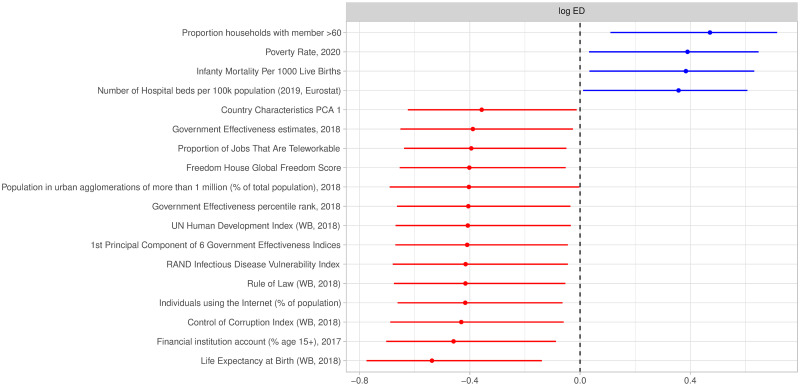
Correlations of country-specific intercepts with country-specific characteristics for excess deaths at 95% credible interval level.

In Fig 5(C) we display the country-specific characteristics that are significantly correlated with excess deaths. We observe higher excess deaths, accounting for interventions, for countries with high proportions of households with members over 60, high poverty rates, high infant mortality and high numbers of hospital beds per 100k population. Conversely, we observe a long list of country characteristics which are negatively correlated with excess deaths, typically associated with developed and rich countries (e.g. life expectancy at birth, control of corruption or government effectiveness).

We describe the effects of the dominant variant and vaccinations in Tables C.4—C.8 of the S1 File. It is important to note that we consider time periods for which there was a single dominant variant, and differences between periods do not reflect epidemiological or phenotypic characteristics of that variant.

Transmission intensity was significantly positively associated with the periods where Delta and Omicron dominated. For the period where Alpha was dominant, there was a negative association with transmission intensity (due to very stringent interventions in place then). Excess deaths had a significant and positive coefficient during the Wildtype period. During the Omicron period, we observe a negative coefficient, but this coefficient needs to be considered cautiously as we only cover a brief time period for omicron. Changes in economic activity are negatively associated with the Wildtype and Alpha periods, and positively for Delta and Omicron periods, indication that the economy adjusted to the pandemic after 2020, with less stringent restrictions as vaccinations were rolled out and individuals adapting more generally to the pandemic [50]. We observed positive coefficients for changes in transit mobility during the Alpha period, as mobility increased over the first half of 2021, and a negative coefficient for omicron driven by reductions in mobility in December 2021.

We estimate a negative association between vaccination and excess deaths consistent with the leading role the vaccination program played in reducing mortality. The lack of significant association between vaccination and economic activity might be linked to the vaccination roll out schedule which focused on the elderly and vulnerable, rather than the working-age population.

## Discussion

The interplay between transmission intensity, excess deaths, population mobility and economic output is highly complex, and none of these factors can be considered in isolation. Here, we study their interaction holistically, for 25 European countries, during the first two years of the SARS-CoV-2 pandemic. Higher transmission intensity decreased GDP and increased excess deaths as can be seen from the impulse responses in Fig 2. Intensified public health interventions reduced mobility, transmission intensity, excess deaths and GDP. Interestingly, because our model allows asymmetric relationships, we found that changes in GDP alone (independent of control measures) had no significant impact on either transmission intensity or excess deaths as we see no impulse response to a shock in GDP (Fig 2). These findings generalise and extend results from previous studies [25], and make them more robust by considering different specifications of our model. Broadly, our results quantify the intuitive phenomenon that significant economic activity arises from person-to-person interaction. Reductions in such interactions—by governmental mandate or behavioural change—reduce transmission, but also harm economic activity.

NPIs affect transmission, GDP and excess deaths in many ways, but act differently to one another. International travel restrictions reduce mortality, but increase economic activity, as more domestic activity can take place where importation of the virus is slowed due to quarantine, testing or outright travel bans [51]. We found, consistent with other work [40], that workplace closures are associated with reduced transmission. We also find that specific economic characteristics of each country reduce transmission intensity. For example, countries with more face-to-face service sectors have higher transmission intensity (see Table C.9 in the S1 File). The effect of NPIs is consistent across each model variant we consider. The association between NPIs and *logR*_*t*_ are consistent with the broader literature [20, 52].

Facial coverings (masks of all types) are an important intervention, as their societal costs and impacts are relatively small and no future economic costs are associated with facial coverings [53]. Economically, facial coverings allow for more open societies, and therefore economic activity (as changes in economic activity and face mask mandates are positively associated). The impact of facial coverings on transmission intensity and excess deaths is more difficult to assess, as this is a function of adherence and face covering quality. We see positive associations between facial coverings (or mask mandates), and both excess deaths and transmission intensity. This may seem counter-intuitive but may be regarded as a consequence of an asymmetric net effect of the lifting of other restrictions that often accompanies increased mask-wearing. This interpretation is consistent with behavioural survey data, such as the Imperial College YouGov COVID Behavioural Tracker survey data [54] which indicates that it takes time for individuals to adhere fully to the face mask mandates at the imposition of the mandate [50] but that individuals are quick to stop using face coverings once mandates are relaxed.

Income support is an important intervention to support economic output but direct interpretation of this coefficient requires caution. Income support itself is a government transfer payment which has no impact on GDP. The positive coefficient with respect to ΔGDP which we observe in the model is the indirect result of two effects. First, income support enables individuals to continue to consume, even if they cannot work. Second, it enables businesses to continue to employ staff and maintain basic functions through the most significant restrictions, so that they can restart activity as soon as possible following lifting of those restrictions.

The behavioural response of individuals is an important factor impacting both disease transmission and economic activity. Fenichel et al [24] used a highly mechanistic model to estimate the impact of behaviour on person-to-person contact and the estimates of *R*_*t*_. The most direct proxy for behaviour in our model is transit mobility and, according to our estimates, individuals reduce their transit mobility as disease transmission and excess deaths increase, exhibiting risk-averse behaviour. Individuals increase their transit mobility in response to increasing economic activity, exhibiting risk-seeking behaviour. Further, individuals reduce their transit mobility in response to NPIs. However, in the longer term, the stringency of restrictions has no statistically significant impact.

Country-specific intercepts in our model account for residual variation, after considering the past dynamics of the response variables, NPIs, vaccination and the variant of SARS-CoV-2 that is currently dominant. Country-specific intercepts for excess deaths and GDP were correlated with several country-specific characteristics (Fig 5), suggesting that more developed European countries had more cautious approaches to the pandemic, prioritising healthcare and lower mortality over economic performance. This can also be seen in Figure C.18 in the S1 File, where there is a positive slope between country-specific intercepts for excess deaths and changes in GDP. Although country-specific intercepts account for latent factors, the collinearity between the trends in GDP and excess deaths suggests a trade-off between them. Additionally, the choice to alter economic performance is not binary and needs to be carefully calibrated according to a country’s economic outlook, policy objectives and fiscal ability to sustain restrictions and associated support.

Several country characteristics are associated with lower excess deaths (Fig 5). Most characteristics reflect the general development level of a country, such as the Global freedom score, Control of Corruption, Internet usage, and the proportion of jobs which are teleworkable.

We find that larger hospital capacity is associated with greater excess deaths. This may seem counter-intuitive but is consistent with previous studies (e.g. Haw et al [5]) which used mechanistic models to explore different scenarios of hospital capacity and the impact on deaths. This association arises because a higher healthcare capacity allows policymakers to delay the introduction of more intensive, but ultimately necessary, NPIs for longer than smaller healthcare capacity would allow, resulting in more infections and deaths. We also note that the capacity and the quality of a healthcare system are not necessarily equivalent, and vary across countries. One limitation of our model is that we cannot quantify the counterfactual effect size from delaying interventions and the commensurate effect on health care capacity. This would be a promising area of future research and likely be only possible to estimate over small regional scales.

A limitation of our analysis is that we do not consider long-term health and economic impacts of the pandemic, such as those caused by loss of schooling [6], mental health impacts [55] and Long COVID [56], as our work is focused on the short term impact of the pandemic over the first 2 years. Another limitation is our assumption of a linear relationship between disease transmission, excess deaths, economic output and transit mobility and other covariates (such as NPIs). More generally, we do not model any latent processes in our analysis—e.g. underlying infection incidence—and we are therefore limited in our ability to mechanistically represent relationships between response variables and interventions. Conversely, our omission of latent processes has the advantage that our model is less constrained by structural assumptions than a more mechanistic modelling framework.

Future research should address the above limitations. In particular, despite the greater structural constraints, more mechanistic representations of both disease epidemiology (e.g. extending the framework of Brauner et al [18] to include economic impacts) and the economy (e.g. by embedding economic variables into the modelling framework) should provide greater insight into the relationships between government policies, country differences and the resulting health and economic effects of the pandemic. The model could be further extended to consider trade as a measure of economic activity, such as in Depero et al [57]. A further extension could be a comparison of the disease spread characteristics to that of other diseases, such as mpox.

## Supporting information

S1 File Supplementary information. This file contains all supplementary results which we have referred to in the main text.
(PDF)
