# Supplementary material for: The interaction of disease transmission, mortality, and economic output over the first 2 years of the COVID-19 pandemic
Source: PLoS One. 2024 Jun 13;19(6):e0301785. doi: 10.1371/journal.pone.0301785 (PMC11175517; doi:10.1371/journal.pone.0301785)
Supplement: S1 File — This file contains all supplementary results which we have referred to in the main text. (PDF) [file pone.0301785.s001.pdf]

# Supplementary Information

## The interaction of disease transmission, mortality, and economic output over the first 2 years of the COVID-19 pandemic

Christian Morgenstern[1,#], Daniel J. Laydon[1], Charles Whittaker[1], Swapnil Mishra[1,2], David Haw[1], Samir Bhatt[1,*], and Neil M. Ferguson[1,*]

[1]MRC Centre for Global Infectious Disease Analysis & WHO Collaborating Centre for Infectious Disease Modelling, Jameel Institute, School of Public Health, Imperial College London, UK
[2]University of Copenhagen, Denmark
[*]Contributed equally
[#] Corresponding author: c.morgenstern@imperial.ac.uk

October 2023

## Contents

**A Pandemic information / data plots** **2**
- A.1 Response Variables, by country, over the full period . . . 2
- A.2 Oxford Covid Government Response - Measures across Containment & Health policies, Economic policies and Health System policies . . . 5
- A.3 Vaccination & Variant data . . . 9
- A.4 GDP Impact . . . 11
- A.5 School Closure data & impact . . . 12
- A.6 Country Characteristic Data . . . 13

**B Further methods** **15**
- B.1 Reproduction Numbers using EpiNow2 . . . 15
- B.2 Model specifications . . . 15
- B.3 Identification strategy . . . 16

**C Estimation results** **17**
- C.1 Model 1 . . . 17
- C.2 Model 2 . . . 17
- C.3 Model 3 . . . 18
- C.4 Country specific characteristics (Model 2) . . . 24
- C.5 Forecast comparison . . . 28
- C.6 Sensitivity Analysis . . . 30
- C.7 Convergence statistics . . . 31

# A Pandemic information / data plots

We consider data for the SARS-CoV-2 pandemic for 25 European countries from 1 Jan 2020 to 31 Dec 2021. The start of the data availability varies by country as a function of the start of the pandemic and data collection in that country.

## A.1 Response Variables, by country, over the full period

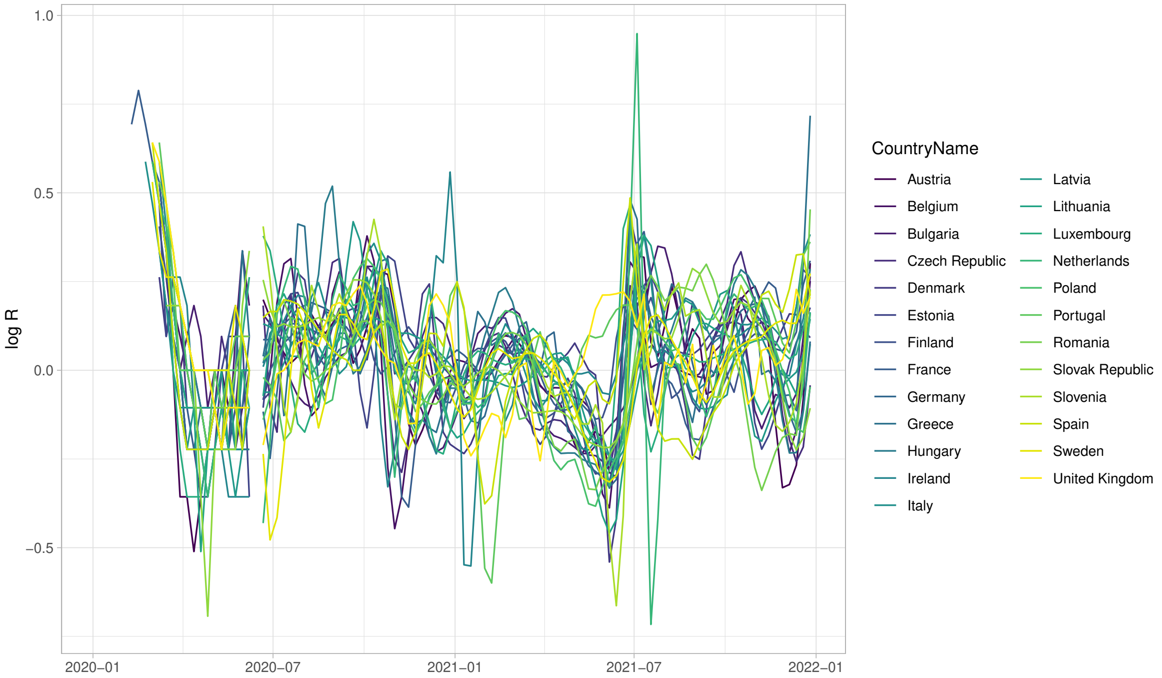


Figure A.1: log R (transmission intensity) over the course of 2020-21. We can observe heterogeneity across both countries and time.

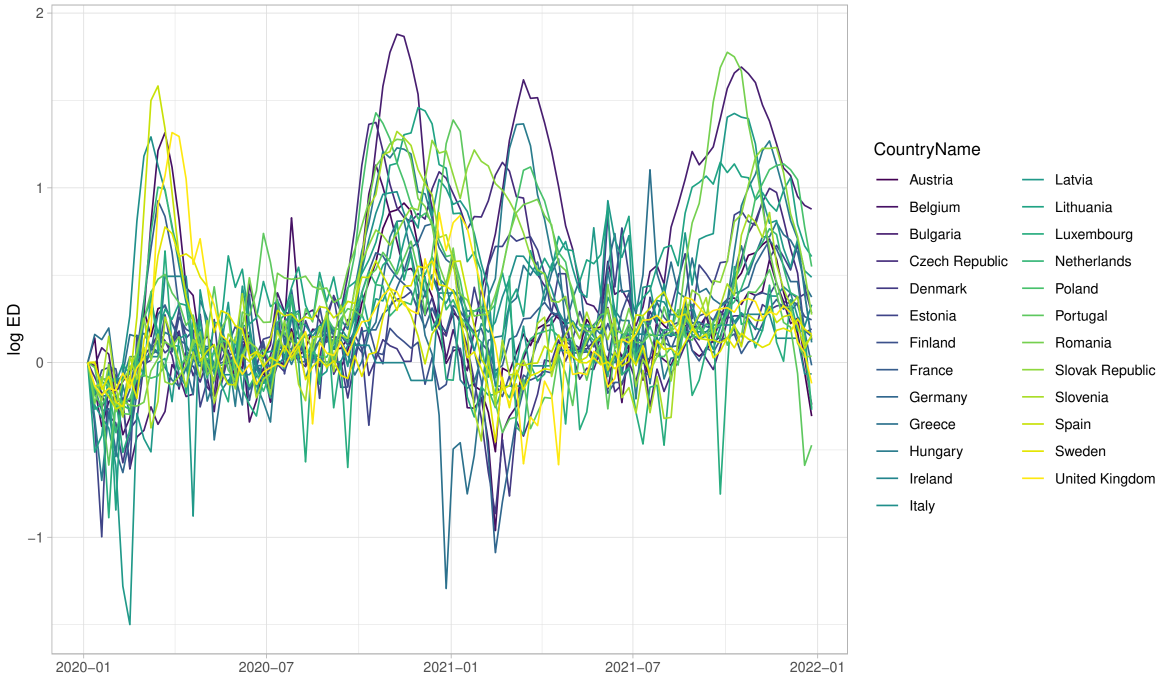


Figure A.2: log excess deaths over the course of 2020-21. We can observe heterogeneity across both countries and time.

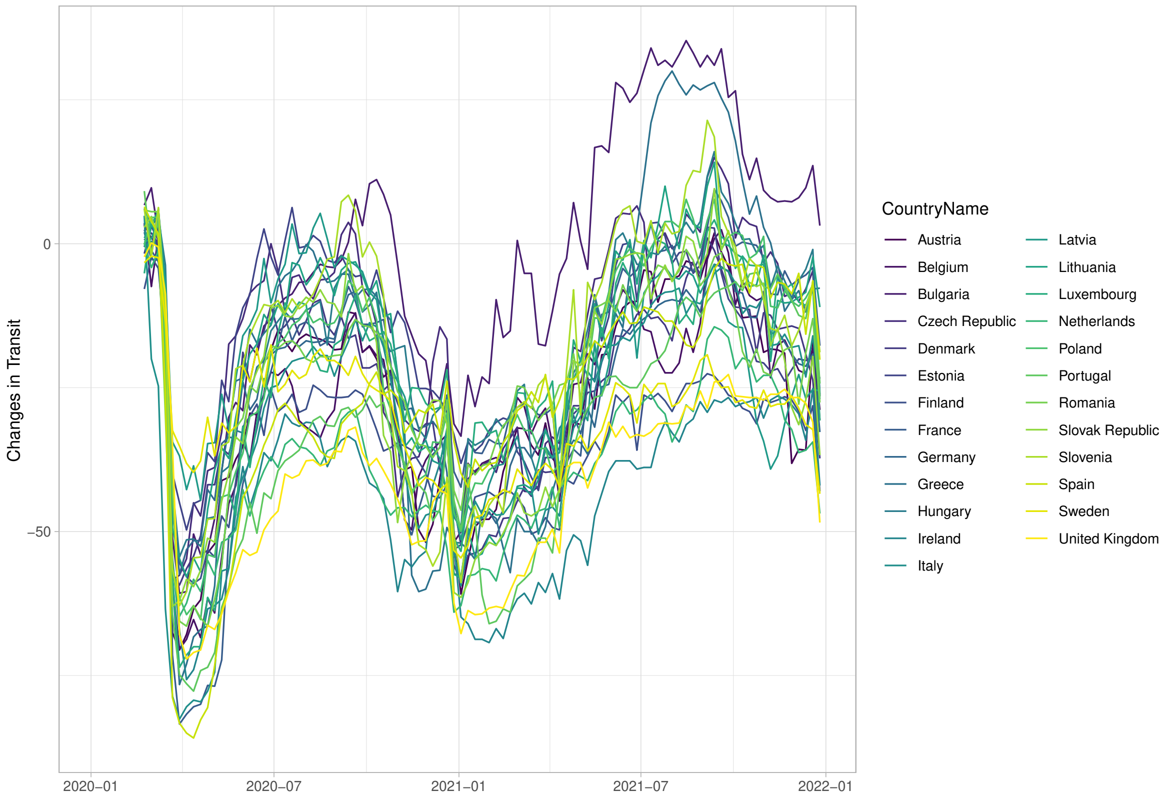


Figure A.3: Changes in transit (Google mobility) over the course of 2020-21. We can observe heterogeneity across both countries and time.

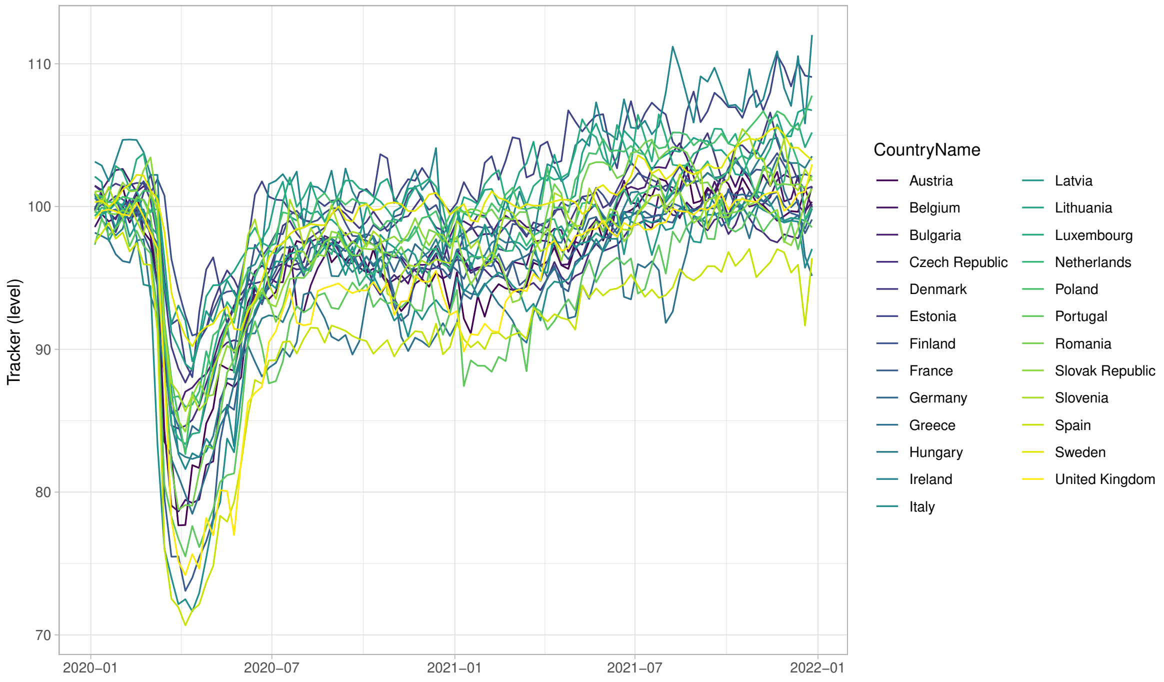


Figure A.4: Indexed GDP over the course of 2020-21. We can observe heterogeneity across both countries and time.

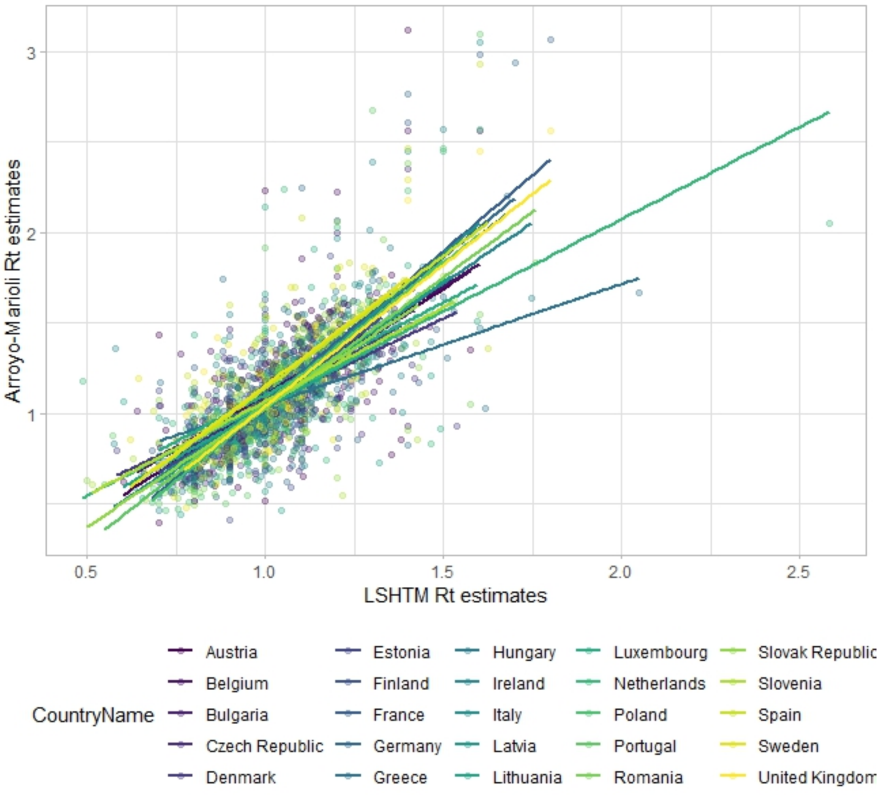


Figure A.5: $R_t$ comparison of [1] and [2]

## A.2 Oxford Covid Government Response - Measures across Containment & Health policies, Economic policies and Health System policies

NPI data is obtained from the Oxford Blavatnik School of Government Covid-19 Government Response Tracker (OxCGRT) [3]. OxCGRT provides systematic data on interventions implemented by governments on a daily basis, since the start of the pandemic. The policy actions are split into 3 categories: 8 containment and closure policy indicators (C1-C8), 4 economic policy indicators (E1-E4) and 8 health system policy indicators (H1-H8). For each measure a score is available that reflects the severity / scale of the restriction, see Tables A.1, A.2, A.3.

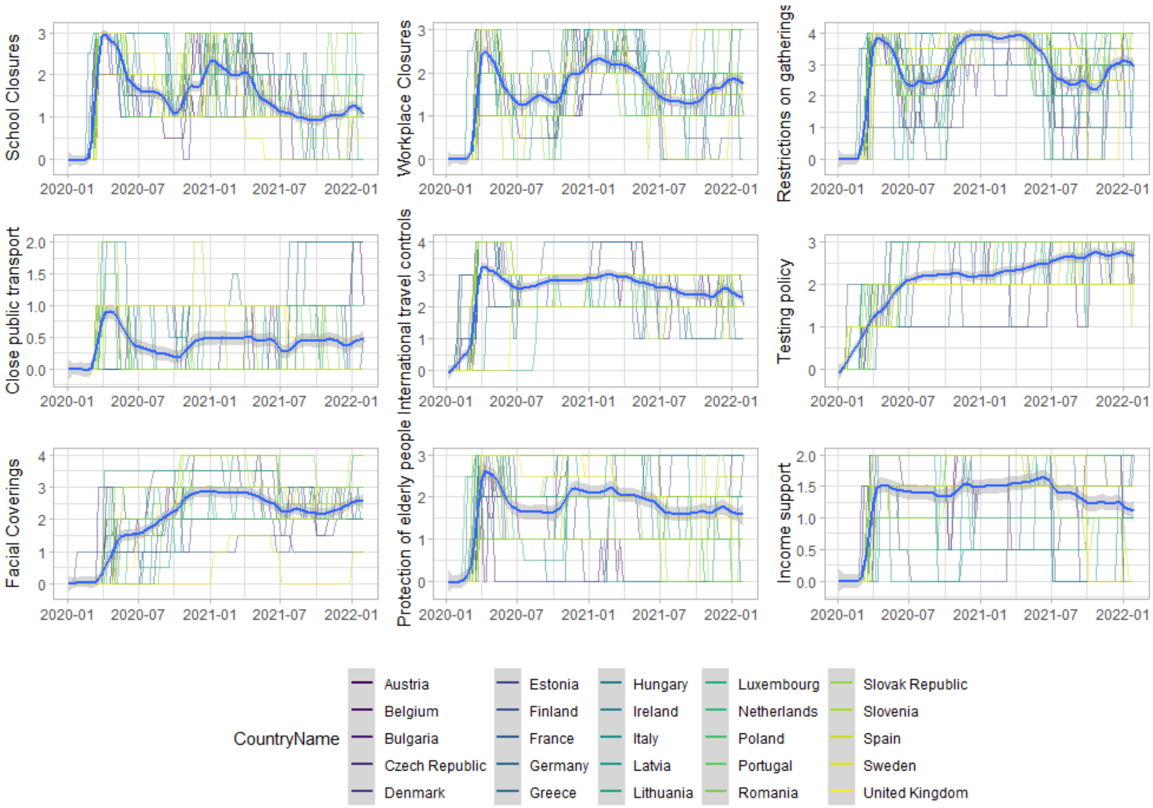


Figure A.6: Non-pharmaceutical Interventions: Time-series of NPIs over the cause of the pandemic by country. The blue line is the smoothed pan-European average for each NPI.

| ID | Name | Description | Coding |
|---|---|---|---|
| C1 | School closing | Record closings of schools and universities | 1 - recommend closing or open with alterations<br>2 - require closing (only some levels or categories)<br>3 - require closing all levels |
| C2 | workplace closure | Record closings of workplaces | 1 - recommend closing or all businesses open with changes<br>2 - require closing (only some levels or categories)<br>3 - require closing for all-but-essential workplaces |
| C3 | Cancel public events | Record cancelling public events | 1 - recommend cancelling<br>2 - require cancelling |
| C4 | Restrictions on gatherings | Record limits on gatherings | 1 - restrictions above 1000 people<br>2 - restrictions between 101-1000 people<br>3 - restrictions between 11-100 people<br>4 - restrictions on 10 people or less |
| C5 | Close public transport | Record closing of public transport | 1 - recommend closing<br>2 - require closing |
| C6 | Stay at home requirement | Record orders to ”shelter-in-place” and otherwise confine to the home | 1 - recommend not leaving house<br>2 - require not leaving house with exceptions |
| C7 | Restrictions on internal movement | Record restrictions on internal movement between cities/regions | 1 - recommend not to travel between regions/cities<br>2 - internal movement restrictions in place<br>3 - require not leaving house with minimal exceptions |
| C8 | International travel controls | Record restrictions on international travel[a] | 1 - screening arrivals<br>2 - quarantine arrivals from some or all regions<br>3 - ban arrivals from some regions<br>4 - ban on all regions or total border closure |

Table A.1: OxCGRT Codebook Containment and closure policies [3]: Missing data will be represented as blank in the database, Coding 0 will be applied if no measure was in place at that moment in time. All Measures are at Ordinal Scale.

[a]Note: this records policy for foreign travellers, not citizens

| ID | Name | Description | Measure | Coding |
|---|---|---|---|---|
| E1 | Income Support | Record if the government is providing direct cash payments to people who lose their jobs or cannot work.[a] | Ordinal scale | 1 - government is replacing less than 50% of lost salary (or if a flat sum, it is less than 50% median salary)<br>2 - government is replacing 50% or more of lost salary (or if a flat sum, it is greater than 50% median salary) |
| E2 | Debt/contract relief | Record if the government is freezing financial obligations for households | Ordinal scale | 1 - narrow relief, specific to one kind of contract<br>2 - broad debt/contract relief |
| E3 | Fiscal measures | Announced economic stimulus spending | USD | Record monetary value in USD of fiscal stimuli, includes any spending or tax cuts NOT included in E4, H4 or H5 |
| E4 | International Support | Announced offers of Covid-19 related aid spending to other countries | USD | Record monetary value in USD |

Table A.2: OxCGRT Codebook economic policies [3]: Missing data will be represented as blank in the database, Coding 0 will be applied if no measure or support was in place at that moment in time.

[a]Note: only includes payments to firms if explicitly linked to payroll/salaries

| ID | Name | Description | Measure | Coding |
|---|---|---|---|---|
| H1 | Public information campaigns | Record presence of public info campaigns | Ordinal scale | 1 - public officials urging caution about Covid-19<br>2 - coordinated public information campaign |
| H2 | Testing policy | Record government policy on who has access to testing | Ordinal scale | 1 - only those who both (a) have symptoms AND (b) meet specific criteria<br>2 - testing of anyone showing Covid-19 symptoms<br>3 - open public testing |
| H3 | Contact tracing | Record government policy on contact tracing after a positive diagnosis[a] | Ordinal scale | 1 - limited contact tracing; not done for all cases<br>2 - comprehensive contact tracing; done for all identified cases |
| H4 | Emergency investment in healthcare | Announced spending on healthcare system [b] | USD | Record monetary value in USD |
| H5 | Investment in vaccines | Announced public spending on Covid-19 vaccine development | USD | Record monetary value in USD |
| H6 | Facial coverings | Record policies on the use of facial coverings outside the home | Ordinal scale | 1 - Recommended<br>2 - Required in some specified shared/public spaces<br>3 - Required in all shared/public spaces<br>4 - Required outside the home at all times |
| H7 | Vaccination policy | Record policies for vaccine delivery for different groups | Ordinal scale | 1 - Availability for ONE group[c]<br>2 - Availability for TWO group<br>3 - Availability for ALL group<br>4 - Available to ALL groups + some others<br>5 - Universally available |
| H8 | Protection of elderly people | Record policies for protecting elderly people in LT Care Facilities/community setting | Ordinal scale | 1 - Recommended restriction measures in LTCFs<br>2 - Narrow restrictions measures in LTCFs<br>3 - Extensive restrictions measures in LTCFs |

Table A.3: OxCGRT Codebook Health system policies [3]: Missing data will be represented as blank in the database, Coding 0 will be applied if no measure was in place at that moment in time.

[a] Note: we are looking for policies that would identify all people potentially exposed to Covid-19; voluntary bluetooth apps are unlikely to achieve this

[b] Note: only record amount additional to previously announced spending

[c] Groups: key workers/ clinically vulnerable groups (non elderly) / elderly groups

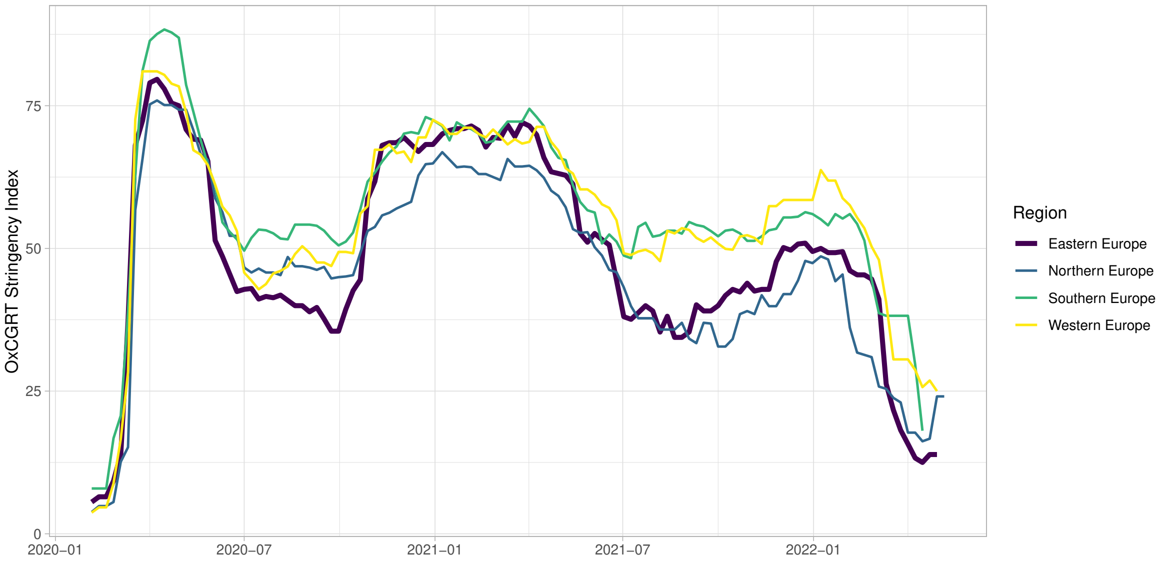


Figure A.7: Oxford Covid Government Responds Tracker NPI Stringency by European sub-region.

## A.3 Vaccination & Variant data

Our World In Data [4] provides a range of vaccination statistics. We use the total number of vaccine doses administered over time. dividing by the total population [5] to calculate the number of doses delivered per capita.

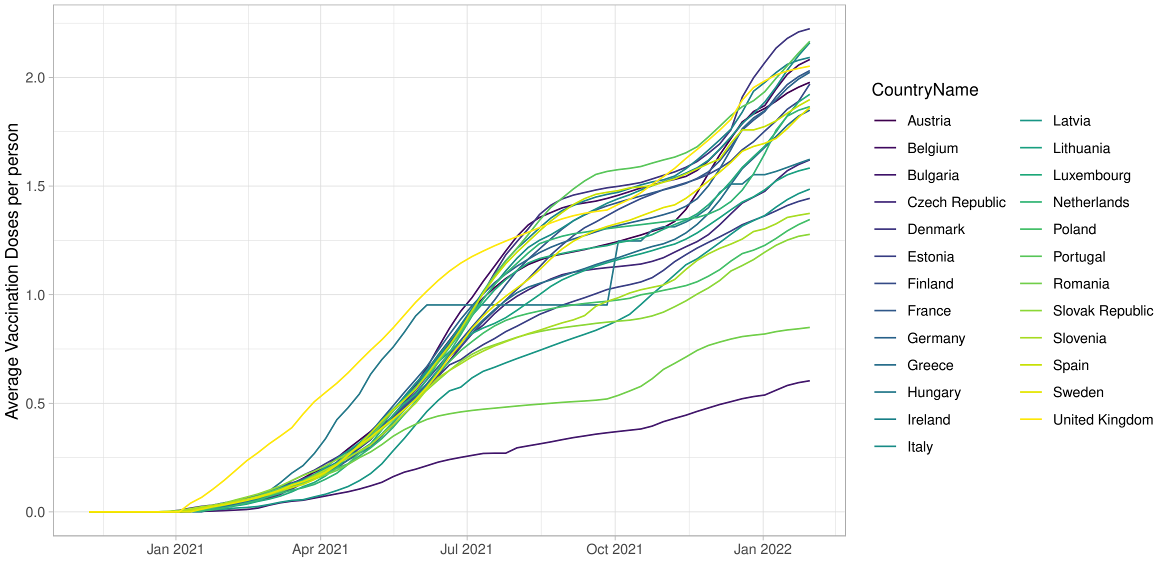


Figure A.8: Vaccinations: Average vaccination per person (total number of vaccines delivered divided by total population).

SARS-CoV-2 variant data is sourced from [6], which provides a summary view by country and week based on GISAID data. We utilise the Nextstrain Clade, Pango Lineage and WHO Label mapping to map all Nextstrain Clades into WHO Labels, for use in the model. For each week we pick the dominant strain in each country as the strain with the majority of sequenced samples (Figure A.9). We observe a data gap for Hungary, which is missing weeks 9-45 in 2021. We estimate data for Hungary during this time period by using the average of the data from surrounding countries, weighted by border length.

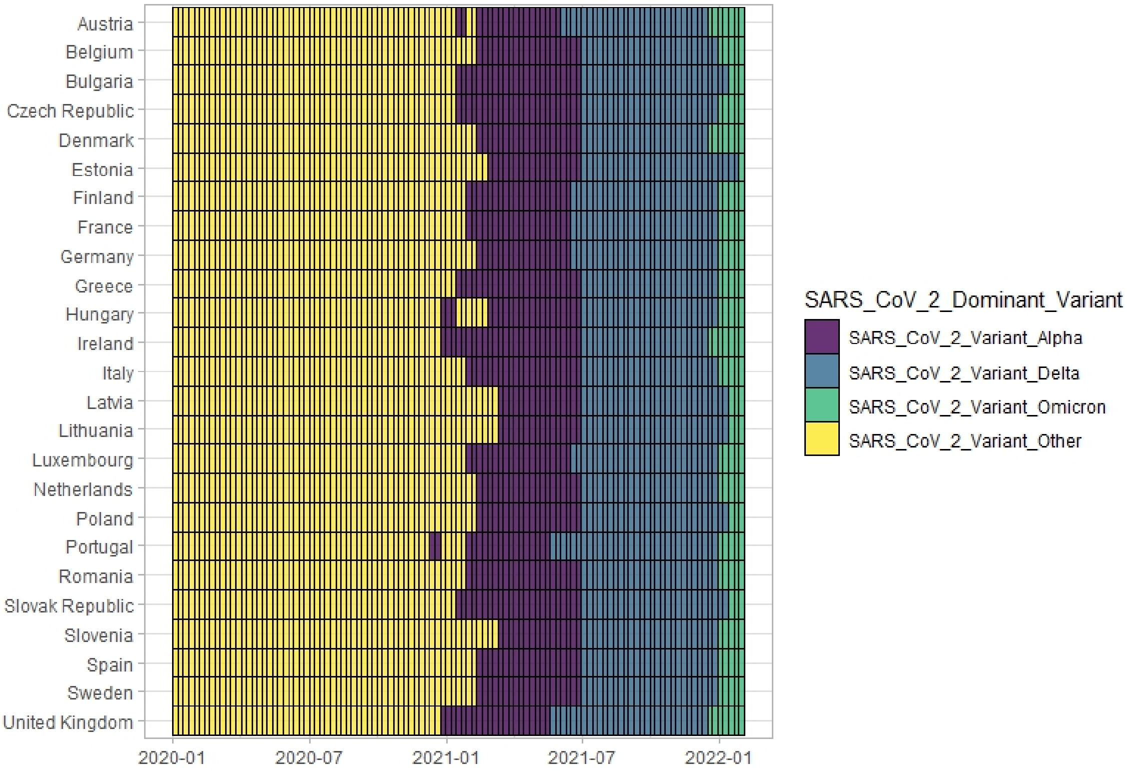


Figure A.9: SARS-CoV-2 Dominant Variant: Largest Variant as measured by sequenced samples over time in each country.

## A.4 GDP Impact

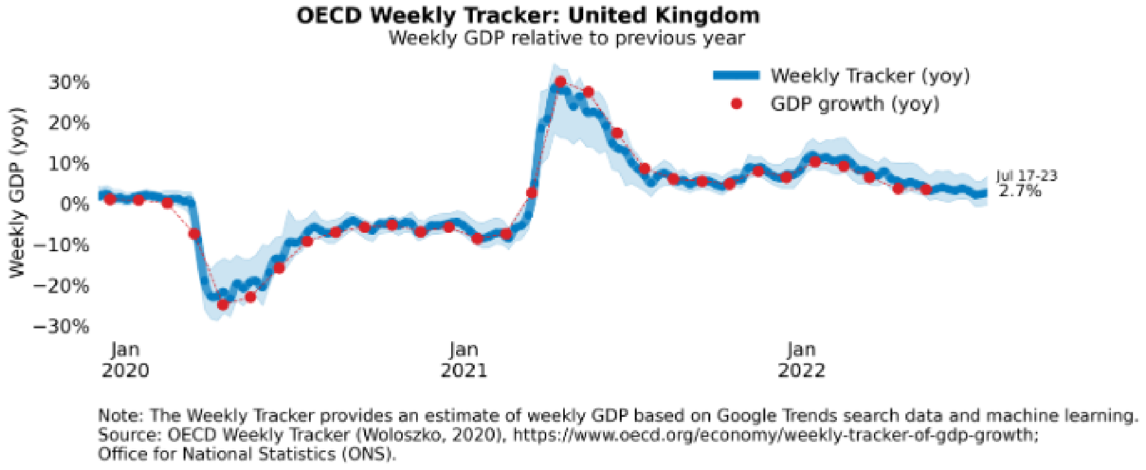


]

Figure A.10: OECD GDP Nowcast validation: Comparsion of GDP Nowcast (YoY) and GDP (YoY) observations from the ONS for the United Kingdom. [Source: OECD Link

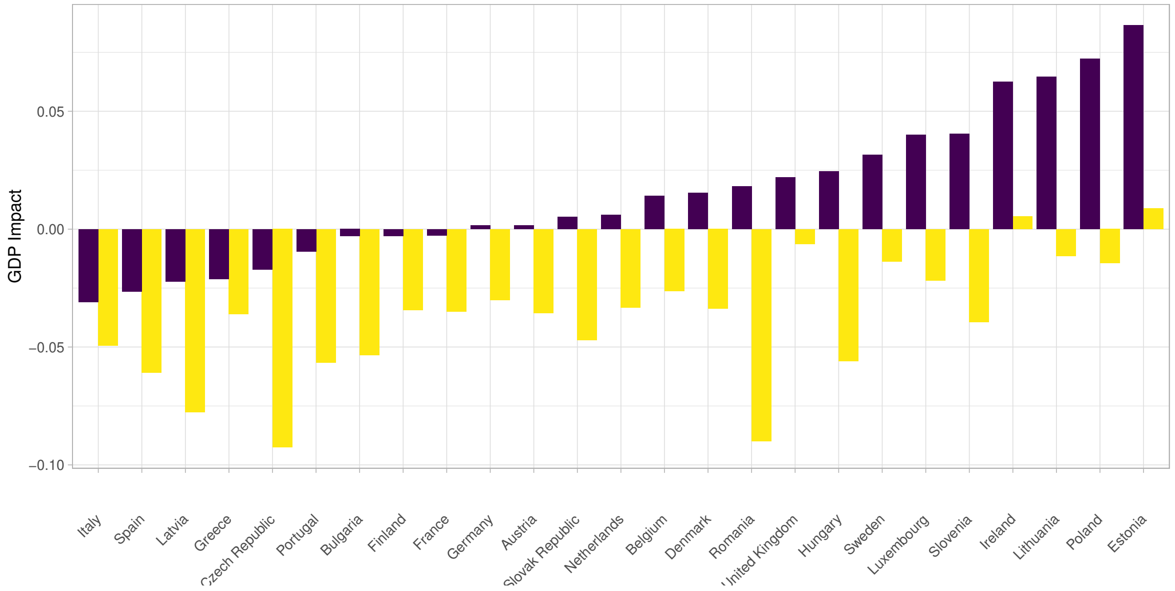


Figure A.11: GDP Impact: We consider the overall GDP growth over the full 2 year period of 2020-21. The purple bars are the observed GDP and the yellow bars are the GDP *including* the counterfactual.

## A.5 School Closure data & impact

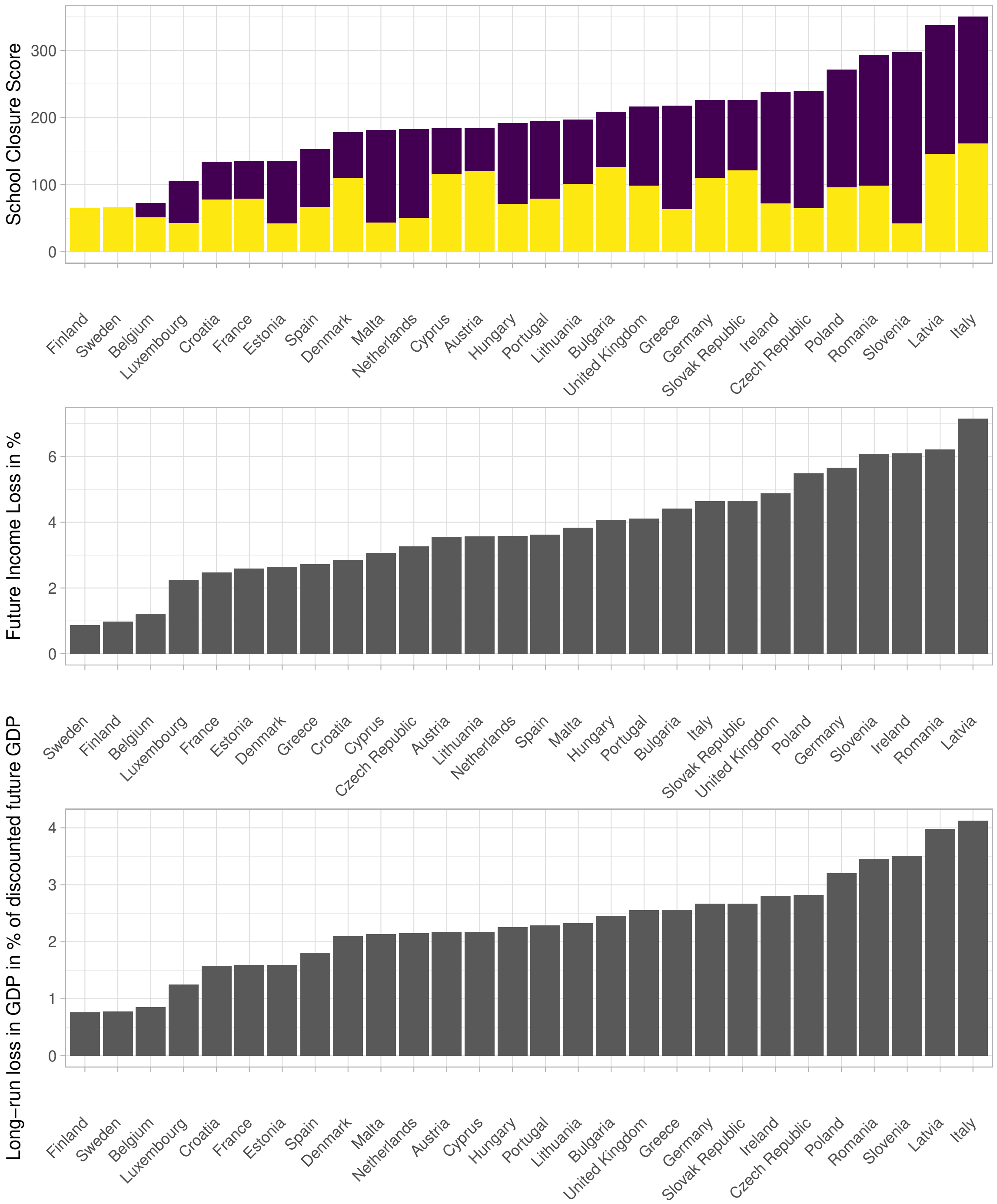


Figure A.12: School Closures: The top panel reports the School Closure score (purple component is equal to the number of days of full school closures, yellow component is a proxy for part shut downs). The middle panel reports the estimated future income loss for impacted students and the bottom panel reports the Long-run loss in GDP as a percentage of discounted future GDP.
*Source:* OECD [7]

## A.6 Country Characteristic Data

We consider a wide range of data in the analysis for which we list a high level overview in Table A.4. This is a mix of time varying variables (such as cases or excess deaths) and variables which we consider time invariant, as they changes over a time frame longer than the time frame of our analysis. We will use these time invariant data as country characteristics in our analysis.

| Data Item | Reference Year / Frequency | Number of Countries | Data Source | Reference |
|---|---|---|---|---|
| **Pandemic Data** | | | | |
| Cases & deaths | daily | 187 | JHU | [3] |
| Hospitalisations | daily | 51 | OWID | [8] |
| Excess deaths | weekly | 29 | Economist | [9] |
| OxCGRT NPIs | daily | 187 | BSG | [3] |
| Vaccination data | daily | 235 | OWID | [8] |
| Phylogenetic data | Weekly | 146 | GISAID | [6] |
| $R_t$ data | daily | 233 | LSHTM | [1] |
| Mobility data | daily | 135 | Google | [10] |
| **Economic Data** | | | | |
| Economic characteristics | 2020 | 17 | ES, WB | [5, 11, 12, 13] |
| Government Finance Stats | annual | 220 | IMF | [14] |
| International Finance Stats | quarterly | 55 | IMF | [14] |
| US Econ data | quarterly | 1 | FED | [15] |
| Economic performance | weekly | 46 | OECD, WB | [16, 11] |
| Electricity data | hourly | 33 | ENTSO-E | [17] |
| Trade flow data | daily | 111 | Comtrade | [18] |
| Pollution data | daily | 153 | OpenAQ | [19] |
| Financial markets data | daily | 67 | BBG | [20] |
| **Government & Health Systems Data** | | | | |
| Government characteristics | 2018 | 24 | WB | [11, 21] |
| Global Freedom Score | 2021 | 25 | FH | [22] |
| Health System data | 2019 | 17 | ES, WB | [5, 11, 23] |
| Mortality data | annual | 155 | ES, WB | [5, 11] |
| **Societal Data** | | | | |
| Societal characteristics | 2017/18 | 25 | WB | [11, 12] |
| YouGov Behavioural surveys | weekly | 30 | YouGov | [24] |
| Political leaning | 2019 | 1 | RCPA3 | [25] |
| Household Composition | 2018 | 155 | UN | [26] |
| Financial Inclusion | 2018 | 155 | WB | [11] |
| Cultural Factors | 2019 | 25 | Paper | [27] |

Table A.4: Country Characteristics: Overview of data items, reference year and coverage of countries. Abbreviations for data sources are EuroStat (ES), Heritage Foundation (HF), World Bank (WB), International Monetary Fund (IMF), United Nations (UN), Freedom House (FH)

In particular we list the country characteristics which we consider in our analysis. We provide the name of the data item (which can be retrieved from the data libraries listed in Table A.4), the latest year for which we have data available and the organisation providing the data:

- % Face-to-Face sectors (2020, Eurostat)
- Number of Physicians per 100k population (2019, Eurostat)
- Number of Hospital beds per 100k population (2019, Eurostat)
- Health Expenditure as Fraction of GDP (2016, WB)
- % of Seats Held by Women in National Parliament (2018, WB)

- Property Rights (2018, Heritage Foundation)
- Regulation Freedom (2018, Heritage Foundation)
- Labor Freedom (2018, Heritage Foundation)
- Freedom House Global Freedom Score (2019, Freedom House)
- RAND Infectious Disease Vulnerability Index (RAND)
- Rule of Law (2018, WB)
- Control of Corruption Index (2018, WB)
- Ages 15-24 Employment to Population Ratio (2018, WB)
- Vulnerable Employment as % of total Employment (2018, WB)
- Ratio of Female to Male Labor Force Participation (2018, WB)
- Life Expectancy at Birth (2018, WB)
- Infanty Mortality Per 1000 Live Births (2018, WB)
- Prevalence of undernourishment (% of population, 2018, WB)
- Net Forest Depletion % of GNI (2018, WB)
- Population Density (2018, WB)
- UN Human Development Index (2018, WB)
- GINI index (2017, WB)
- Access to electricity (% of population) (2018, WB)
- Electric power consumption (kWh per capita) (2014, WB)
- Individuals using the Internet (% of population) (WB)
- Government Effectiveness estimates (2018, WB)
- Financial institution account (% age 15+) (2017, WB)
- Proportion households with member ¿60 (2009-2018, UN)
- Proportion households with member ¡20 and ¿60 (2009-2018, UN)
- Average number of people in household (2009-2018, UN)
- Multi-generation household percent (2009-2018, UN)
- Three-generational household percent (2009-2018, UN)
- Population density (people per sq. km of land area) (2018, WB)
- Urban population (% of total population) (2018, WB)
- Rural population (% of total population) (2017,WB)
- Population living in slums (% of urban population) (2014, WB)
- Population in urban agglomerations of more than 1 million (% of total population) (2018, WB)
- Population in the largest city (% of urban population) (2018, WB)
- Poverty Rate (2020, Lakner et al [12])
- Proportion of Jobs That Are Teleworkable (2020, Dingel et al [13])

# B Further methods

## B.1 Reproduction Numbers using EpiNow2

We use instantaneous reproduction numbers computed by the London School of Hygine and Tropical Medicine (LSHTM) using the EpiNow2 model [1] (the model and code is freely available on github and epiforecasts).

## B.2 Model specifications

We consider 3 model specifications. Model 2 is described in the main text and results are provided. The estimation results for model 1 and 3 are provided in this supplement.

***Model 1*** First we consider the interaction of transmission intensity, excess deaths, changes in NPIs, changes in GDP and changes in transit behaviour. Changes in NPIs are given by changes to the overall stringency index for each country over the respective period of time. The only covariates are vaccination, given by the average number of vaccinations per person for a given country at time $t$ and the dominant variant of SARS-CoV-2.

In this model the predictor coefficients are common across countries, and we have country specific intercepts (random effects) $\mu_c$ for each country $c$.

$$\begin{aligned} Y_{t,c} &\sim \texttt{MVN}\big(y_{t,c}, \Sigma_u\big) \qquad (1) \\ y_{t,c} &= \mu_{\mathbf{c}} + \sum_{k=1}^{p} \Phi_k y_{t-k,c} + \\ &\quad \nu \cdot vacc_{t,c} + \psi \cdot \sum_{\substack{j \in \{WT, Alpha, \\ Delta, Omicron\}}} \Psi_{j,t,c} \\ where:& \\ y_{t,c} &= \big(y_{1t,c}, y_{2t,c}, y_{3t,c}, y_{4t,c}\big)^{\top} \\ &= \big(\log \texttt{R}_t, \log \texttt{Excess Deaths}_t, \Delta\texttt{NPI}_t, \Delta\texttt{GDP}_t, \Delta\texttt{Transit}_t, \big)^{\top} \qquad (2) \\ vacc_t &= \texttt{Average Vaccinations per person}_t \end{aligned}$$

$Y_{t,c}$ is a multivariate normal random variable with covariance matrix $\Sigma_u$. $y_{t,c}$ is a vector containing the values of the response variables, as defined in Equation2, at time $t$ for country $c$. $\Phi_k$ is the $N \times N$ coefficient matrix of the vector auto-regressive component for lag $k$. $\nu$ is the coefficient for vaccination (defined as the average number of vaccinations per person at time $t$ for country $c$). $\Psi_{j,t,c}$ is the coefficient for the $j^{th}$ dominant variant of SARS-CoV-2, at time $t$ for country $c$. $\Psi$ is constructed such that $\Psi_{WT,\cdot,\cdot}$ is always 1 (and acts as an intercept term), $\Psi_{Alpha,\cdot,\cdot}$ is 1 unless Wildtype is the dominant SARS-CoV-2 variant (in which case it is 0), $\Psi_{Delta,\cdot,\cdot}$ is 1 unless Wildtype or AlpgaAlpha are the dominant variants and $\Psi_{Omicron,\cdot,\cdot}$ is 1 only if Omicron is the dominant variant.

***Model 2*** The second model removes stringency from the response variables, and adds individual NPIs (across containment & closures, economic and health system policies) as explanatory variables to the model $x_t$.

Here we consider individual NPIs to be covariates to the response variables. The implementation of NPIs, both in timing and level of severity, have varied between countries. Early in the pandemic (March to September 2020) implementations of mandates were reasonably homogeneous, but we subsequently observe significant heterogeneity, both in time and amongst countries as we observed divergent objectives by policy makers and debates in society on mandates. In this model we consider *lagged* NPIs.

$$
\begin{aligned}
Y_{t,c} &\sim \mathtt{MVN}\big(y_{t,c}, \Sigma_u\big) \qquad (3)\\
y_{t,c} &= \mu_{\mathbf{c}} + \sum_{k=1}^{p} \Phi_k y_{t-k,c} + \lambda \cdot x_{t,c} + \delta \cdot \Delta x_{t,c} + \\
&\quad \nu \cdot vacc_{t,c} + \psi \cdot \sum_{\substack{j \in \{WT, Alpha,\\ Delta, Omicron\}}} \Psi_{j,t,c}\\
where:&\\
y_t &= \big(y_{1t,c}, y_{2t,c}, y_{3t,c}, y_{4t,c}\big)^{\top}\\
&= \big(\log \mathtt{R}_t, \log \mathtt{Excess\ Deaths}_t, \Delta\mathtt{GDP}_t, \Delta\mathtt{Transit}_t, \big)^{\top}\\
x_t &= \mathtt{NPI}_{t-1}\\
vacc_t &= \mathtt{Average\ Vaccinations\ per\ person}_t
\end{aligned}
$$

In addition to the coefficients used in Model 1, we have additional coefficients $\lambda$ and $\delta$, that respectively denote the (fixed-effects) level and changes in NPIs.

***Model 3*** Finally we consider a model with only one response variable and consider the impact of NPIs, vaccination and dominant variant only. This type of model can be considered a simple extension to [28, 29].

$$
\begin{aligned}
Y_{t,c} &\sim \mathtt{N}\big(y_{t,c}, \sigma_u^2\big) \qquad (4)\\
y_{t,c} &= \mu_{\mathbf{c}} + \sum_{k=1}^{p} \phi_k y_{t-k,c} + \lambda \cdot x_{t,c} + \delta \cdot \Delta x_{t,c} + \\
&\quad \nu \cdot vacc_{t,c} + \psi \cdot \sum_{\substack{j \in \{WT, Alpha,\\ Delta, Omicron\}}} \Psi_{j,t,c}\\
where:&\\
x_t &= \mathtt{NPI}_{t-1}\\
vacc_t &= \mathtt{Average\ Vaccinations\ per\ person}_t
\end{aligned}
$$

### B.3 Identification strategy

Example for $N = 2$:

$$
\begin{pmatrix} \sigma_{11}^2 & \sigma_{12}^2 \\ \sigma_{12}^2 & \sigma_{22}^2 \end{pmatrix} = \begin{pmatrix} b_{11}^2 + b_{12}^2 & b_{11}b_{12} + b_{12}b_{22} \\ b_{11}b_{12} + b_{12}b_{22} & b_{12}^2 + b_{22}^2 \end{pmatrix} \qquad (5)
$$

where we need to solve for four unknowns but only have 3 equations.

# C Estimation results

We provide an overview of results for all 3 models in this section. All results can be reproduced with the data and code available on GitHub (`https://github.com/cm401/covid_eco_epi_var`).

## C.1 Model 1

For Model 1 we focus on the results of the Orthogonal Impulse Response Function which we provide in Figure C.13. A positive impulse in transmission intensity leads to an increase in excess deaths, peaking after 5 weeks, and the decay of the impulse is slow and prolonged. A shorter but positive response can also be seen in changes to the overall stringency of NPIs. We also observe a significant and negative response in economic activity but no significant impact on changes in transit behaviour. The effect to a positive impulse in excess deaths is smaller than that for transmission intensity. A positive impulse to changes in the overall stringency of NPIs has only a positive effect on itself but no other variable. Considering economic activity, we observe that a positive impulse leads to negative changes in overall stringency of NPIs, a positive effect on economic activity itself and a significant positive effect on transit mobility in the next time period.

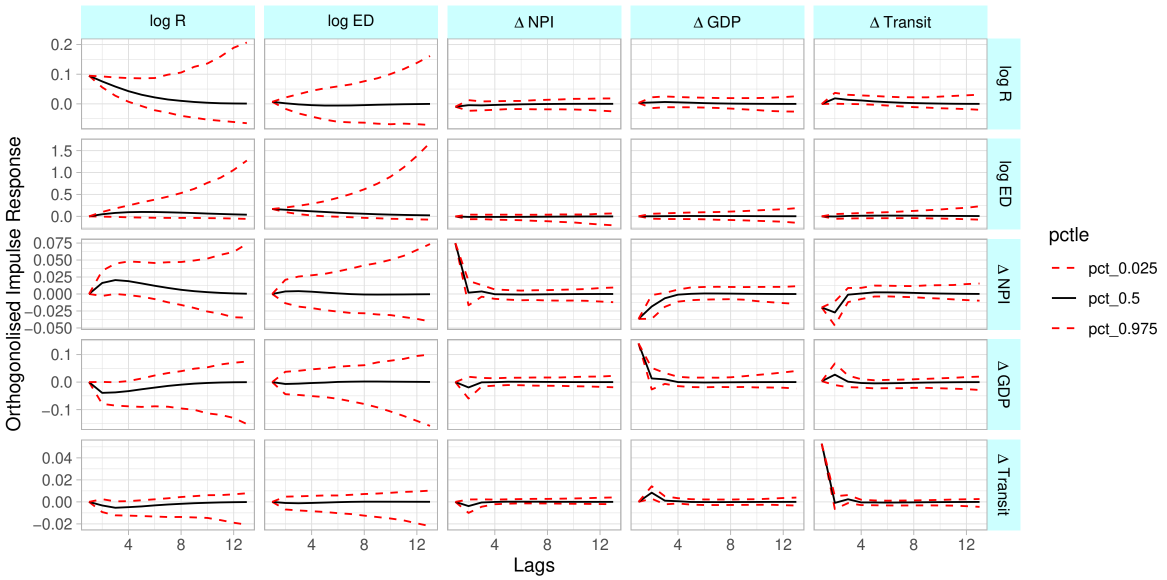


Figure C.13: Model 1 Orthogonal IRF: Columns are the variables to which the shock is applied to, rows are the variables which we observe the impulse response for. Mean estimates (solid black) and 95% CrI (dotted red).

## C.2 Model 2

We provide the coefficient estimates for the model in 5 tables:

1. Estimates of the VAR coefficients $\Phi_{i,j,k=1}$ in Table C.5
2. Estimates of the coefficients for NPI levels $\lambda$ in Table C.6
3. Estimates of the coefficients for NPI changes $\delta$ in Table C.7
4. Estimates for the Dominant Varient coefficients $\psi$ in Table C.8
5. Estimates for Vaccination coefficients $\nu$ in Table C.9

In the results section of the main text we considered the model choice for the specification of Model 2 with

- NPI changes and levels

| Variable | Estimate | Est.Error | CrI Lower | CrI Upper |
|---|---|---|---|---|
| log R, log R l1 | 0.757 | 0.017 | 0.725 | 0.790 |
| log R, log ED l1 | -0.040 | 0.007 | -0.054 | -0.026 |
| log R, Δ GDP l1 | 0.003 | 0.017 | -0.032 | 0.036 |
| log R, Δ Transit l1 | 0.103 | 0.043 | 0.019 | 0.187 |
| log ED, log R l1 | 0.271 | 0.030 | 0.213 | 0.329 |
| log ED, log ED l1 | 0.856 | 0.014 | 0.828 | 0.884 |
| log ED, Δ GDP l1 | -0.008 | 0.031 | -0.067 | 0.053 |
| log ED, Δ Transit l1 | 0.014 | 0.076 | -0.135 | 0.163 |
| Δ GDP, log R l1 | -0.241 | 0.027 | -0.295 | -0.189 |
| Δ GDP, log ED l1 | -0.054 | 0.012 | -0.078 | -0.030 |
| Δ GDP, Δ GDP l1 | 0.046 | 0.028 | -0.008 | 0.100 |
| Δ GDP, Δ Transit l1 | 0.067 | 0.069 | -0.067 | 0.203 |
| Δ Transit, log R l1 | -0.055 | 0.010 | -0.074 | -0.036 |
| Δ Transit, log ED l1 | -0.025 | 0.004 | -0.033 | -0.018 |
| Δ Transit, Δ GDP l1 | 0.135 | 0.010 | 0.115 | 0.156 |
| Δ Transit, Δ Transit l1 | -0.113 | 0.025 | -0.163 | -0.063 |

Table C.5: Estimates for VAR coefficients $\Phi_{i,j,k=1}$ of the model

- NPI changes
- NPI levels

We can run the model with exactly the same setup but use only NPI changes (LHS of Figure C.14) or use only NPI levels (RHS of Figure C.14). Tables with the numeric values of these estimates can be generated from the model output using the GitHub code.

## C.3 Model 3

For Model 3 we do not have vector auto-regressive coefficients (although we do have auto-regressive coefficients for each one of our response variables). The results for the effect size of NPIs on each of the response variables is displayed in Figure C.15. We observe that estimates are different from Model 2 but substantively the same (sign of estimates are mostly the same and the factors which are significant are broadly similar).

| Variable | Estimate | Est.Error | CrI Lower | CrI Upper |
|---|---|---|---|---|
| log R lvl Schools Closing | -0.006 | 0.004 | -0.014 | 0.002 |
| log R lvl workplace closure | -0.012 | 0.005 | -0.021 | -0.003 |
| log R lvl Restrictions on gatherings | -0.006 | 0.003 | -0.012 | -0.001 |
| log R lvl Close public transport | 0.007 | 0.005 | -0.003 | 0.017 |
| log R lvl International travel controls | 0.003 | 0.004 | -0.004 | 0.011 |
| log R lvl Testing policy | 0.007 | 0.005 | -0.003 | 0.016 |
| log R lvl Facial Coverings | 0.010 | 0.003 | 0.004 | 0.015 |
| log R lvl Protection of elderly people | -0.007 | 0.003 | -0.013 | -0.000 |
| log R lvl Income support | 0.002 | 0.004 | -0.006 | 0.010 |
| log ED lvl Schools Closing | -0.010 | 0.008 | -0.026 | 0.006 |
| log ED lvl workplace closure | -0.002 | 0.009 | -0.019 | 0.015 |
| log ED lvl Restrictions on gatherings | 0.002 | 0.005 | -0.008 | 0.012 |
| log ED lvl Close public transport | 0.005 | 0.010 | -0.015 | 0.024 |
| log ED lvl International travel controls | -0.014 | 0.007 | -0.028 | -0.001 |
| log ED lvl Testing policy | -0.005 | 0.009 | -0.023 | 0.013 |
| log ED lvl Facial Coverings | 0.011 | 0.005 | 0.000 | 0.021 |
| log ED lvl Protection of elderly people | -0.001 | 0.006 | -0.014 | 0.011 |
| log ED lvl Income support | -0.007 | 0.008 | -0.023 | 0.009 |
| $\Delta$ GDP lvl Schools Closing | 0.007 | 0.007 | -0.007 | 0.020 |
| $\Delta$ GDP lvl workplace closure | 0.002 | 0.008 | -0.013 | 0.018 |
| $\Delta$ GDP lvl Restrictions on gatherings | 0.004 | 0.005 | -0.005 | 0.013 |
| $\Delta$ GDP lvl Close public transport | -0.001 | 0.009 | -0.018 | 0.016 |
| $\Delta$ GDP lvl International travel controls | 0.014 | 0.006 | 0.002 | 0.025 |
| $\Delta$ GDP lvl Testing policy | 0.007 | 0.008 | -0.008 | 0.024 |
| $\Delta$ GDP lvl Facial Coverings | 0.016 | 0.005 | 0.007 | 0.026 |
| $\Delta$ GDP lvl Protection of elderly people | 0.015 | 0.006 | 0.005 | 0.027 |
| $\Delta$ GDP lvl Income support | 0.023 | 0.008 | 0.009 | 0.039 |
| $\Delta$ Transit lvl Schools Closing | 0.001 | 0.002 | -0.003 | 0.006 |
| $\Delta$ Transit lvl workplace closure | -0.000 | 0.003 | -0.006 | 0.005 |
| $\Delta$ Transit lvl Restrictions on gatherings | 0.002 | 0.002 | -0.001 | 0.005 |
| $\Delta$ Transit lvl Close public transport | 0.001 | 0.003 | -0.005 | 0.007 |
| $\Delta$ Transit lvl International travel controls | 0.003 | 0.002 | -0.002 | 0.007 |
| $\Delta$ Transit lvl Testing policy | -0.002 | 0.003 | -0.008 | 0.003 |
| $\Delta$ Transit lvl Facial Coverings | 0.001 | 0.002 | -0.002 | 0.005 |
| $\Delta$ Transit lvl Protection of elderly people | 0.002 | 0.002 | -0.002 | 0.006 |
| $\Delta$ Transit lvl Income support | 0.002 | 0.003 | -0.003 | 0.006 |

Table C.6: Estimates for NPI Level ($\lambda$) coefficients

| Variable | Estimate | Est.Error | CrI Lower | CrI Upper |
|---|---|---|---|---|
| log R Δ Schools Closing | -0.003 | 0.007 | -0.017 | 0.011 |
| log R Δ workplace closure | -0.011 | 0.008 | -0.026 | 0.005 |
| log R Δ Restrictions on gatherings | -0.004 | 0.005 | -0.014 | 0.006 |
| log R Δ Close public transport | -0.013 | 0.014 | -0.040 | 0.015 |
| log R Δ International travel controls | 0.007 | 0.008 | -0.008 | 0.022 |
| log R Δ Testing policy | -0.003 | 0.011 | -0.024 | 0.018 |
| log R Δ Facial Coverings | -0.004 | 0.009 | -0.022 | 0.013 |
| log R Δ Protection of elderly people | -0.000 | 0.007 | -0.015 | 0.013 |
| log R Δ Income support | 0.014 | 0.011 | -0.008 | 0.037 |
| log ED Δ Schools Closing | -0.007 | 0.013 | -0.032 | 0.019 |
| log ED Δ workplace closure | 0.005 | 0.014 | -0.023 | 0.033 |
| log ED Δ Restrictions on gatherings | 0.021 | 0.009 | 0.003 | 0.039 |
| log ED Δ Close public transport | -0.001 | 0.025 | -0.052 | 0.049 |
| log ED Δ International travel controls | -0.026 | 0.014 | -0.053 | 0.001 |
| log ED Δ Testing policy | 0.005 | 0.020 | -0.033 | 0.043 |
| log ED Δ Facial Coverings | -0.003 | 0.016 | -0.034 | 0.028 |
| log ED Δ Protection of elderly people | 0.020 | 0.013 | -0.005 | 0.045 |
| log ED Δ Income support | 0.003 | 0.021 | -0.038 | 0.044 |
| Δ GDP Δ Schools Closing | -0.026 | 0.012 | -0.049 | -0.003 |
| Δ GDP Δ workplace closure | -0.002 | 0.013 | -0.027 | 0.022 |
| Δ GDP Δ Restrictions on gatherings | 0.001 | 0.008 | -0.015 | 0.016 |
| Δ GDP Δ Close public transport | -0.017 | 0.023 | -0.061 | 0.028 |
| Δ GDP Δ International travel controls | -0.023 | 0.012 | -0.047 | 0.001 |
| Δ GDP Δ Testing policy | -0.022 | 0.017 | -0.056 | 0.012 |
| Δ GDP Δ Facial Coverings | 0.005 | 0.014 | -0.023 | 0.032 |
| Δ GDP Δ Protection of elderly people | -0.049 | 0.011 | -0.071 | -0.027 |
| Δ GDP Δ Income support | -0.007 | 0.018 | -0.043 | 0.028 |
| Δ Transit Δ Schools Closing | -0.019 | 0.004 | -0.028 | -0.011 |
| Δ Transit Δ workplace closure | -0.026 | 0.005 | -0.036 | -0.017 |
| Δ Transit Δ Restrictions on gatherings | -0.004 | 0.003 | -0.009 | 0.002 |
| Δ Transit Δ Close public transport | -0.017 | 0.008 | -0.033 | -0.000 |
| Δ Transit Δ International travel controls | -0.030 | 0.005 | -0.038 | -0.021 |
| Δ Transit Δ Testing policy | 0.003 | 0.006 | -0.013 | 0.008 |
| Δ Transit Δ Facial Coverings | -.003 | 0.005 | -0.013 | 0.008 |
| Δ Transit Δ Protection of elderly people | -0.007 | 0.004 | -0.015 | 0.001 |
| Δ Transit Δ Income support | -0.004 | 0.007 | -0.018 | 0.009 |

Table C.7: Estimates for NPI Changes ($\delta$) coefficients

| Variable | Estimate | Est.Error | CrI Lower | CrI Upper |
|---|---|---|---|---|
| log R WT variant | 0.032 | 0.018 | -0.004 | 0.068 |
| log R Alpha variant | -0.027 | 0.008 | -0.042 | -0.012 |
| log R Delta variant | 0.033 | 0.013 | 0.007 | 0.059 |
| log R Omicron variant | 0.090 | 0.034 | 0.022 | 0.156 |
| log ED WT variant | 0.090 | 0.034 | 0.022 | 0.156 |
| log ED Alpha variant | 0.018 | 0.014 | -0.010 | 0.045 |
| log ED Delta variant | 0.024 | 0.024 | -0.024 | 0.071 |
| log ED Omicron variant | -0.129 | 0.067 | -0.259 | 0.003 |
| Δ GDP WT variant | -0.143 | 0.035 | -0.214 | -0.077 |
| Δ GDP Alpha variant | -0.040 | 0.013 | -0.065 | -0.015 |
| Δ GDP Delta variant | 0.093 | 0.021 | 0.050 | 0.135 |
| Δ GDP Omicron variant | 0.115 | 0.059 | 0.002 | 0.230 |
| Δ Transit WT variant | -0.013 | 0.011 | -0.034 | 0.008 |
| Δ Transit Alpha variant | 0.013 | 0.005 | 0.004 | 0.022 |
| Δ Transit Delta variant | 0.015 | 0.008 | -0.001 | 0.030 |
| Δ Transit Omicron variant | -0.050 | 0.021 | -0.092 | -0.009 |

Table C.8: Estimates for Dominant Variant coefficients ($\nu$) coefficients

| Variable | Estimate | Est.Error | CrI Lower | CrI Upper |
|---|---|---|---|---|
| log R vaccination | -0.004 | 0.012 | -0.027 | 0.020 |
| log ED vaccination | -0.047 | 0.023 | -0.092 | -0.002 |
| Δ GDP vaccination | -0.028 | 0.020 | -0.068 | 0.012 |
| Δ Transit vaccination | -0.020 | 0.007 | -0.033 | -0.005 |

Table C.9: Estimates for Vaccination, ($\psi$) coefficients

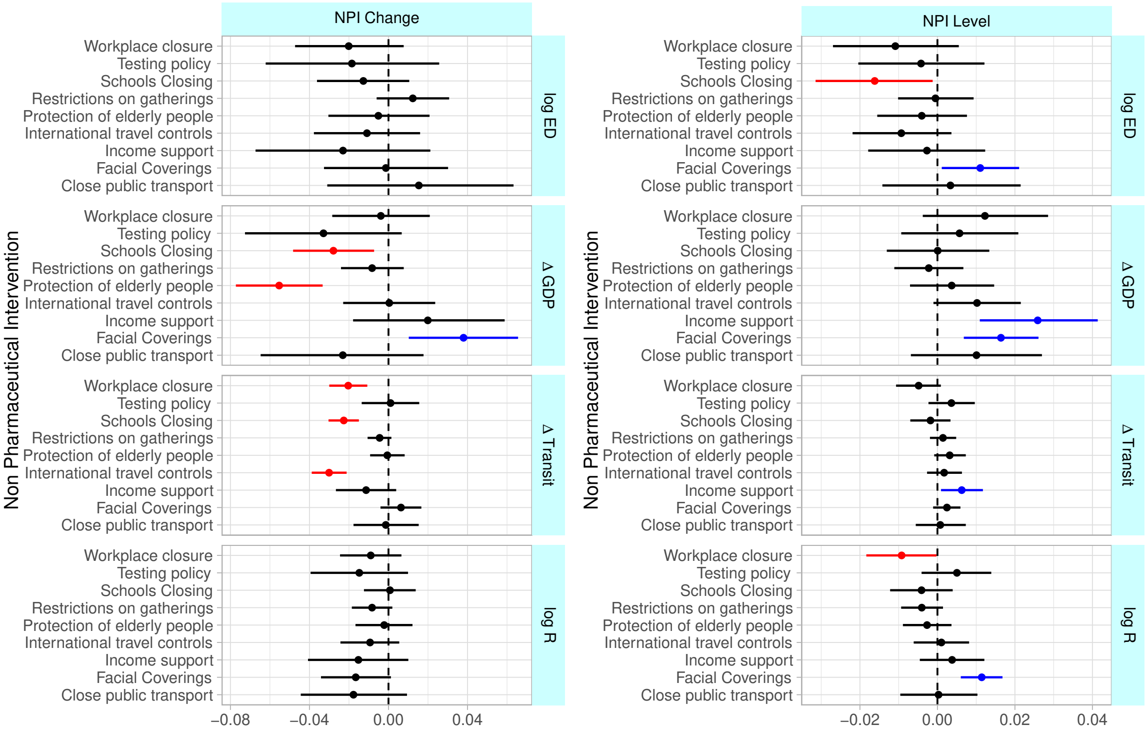


Figure C.14: NPI effect sizes for Model 2 with only changes (LHS) and levels (RHS): Coefficient effect sizes (with 95% credible intervals) for each response variable. Blue highlighted results indicate positive and significant coefficients and red indicates negative and significant coefficients.

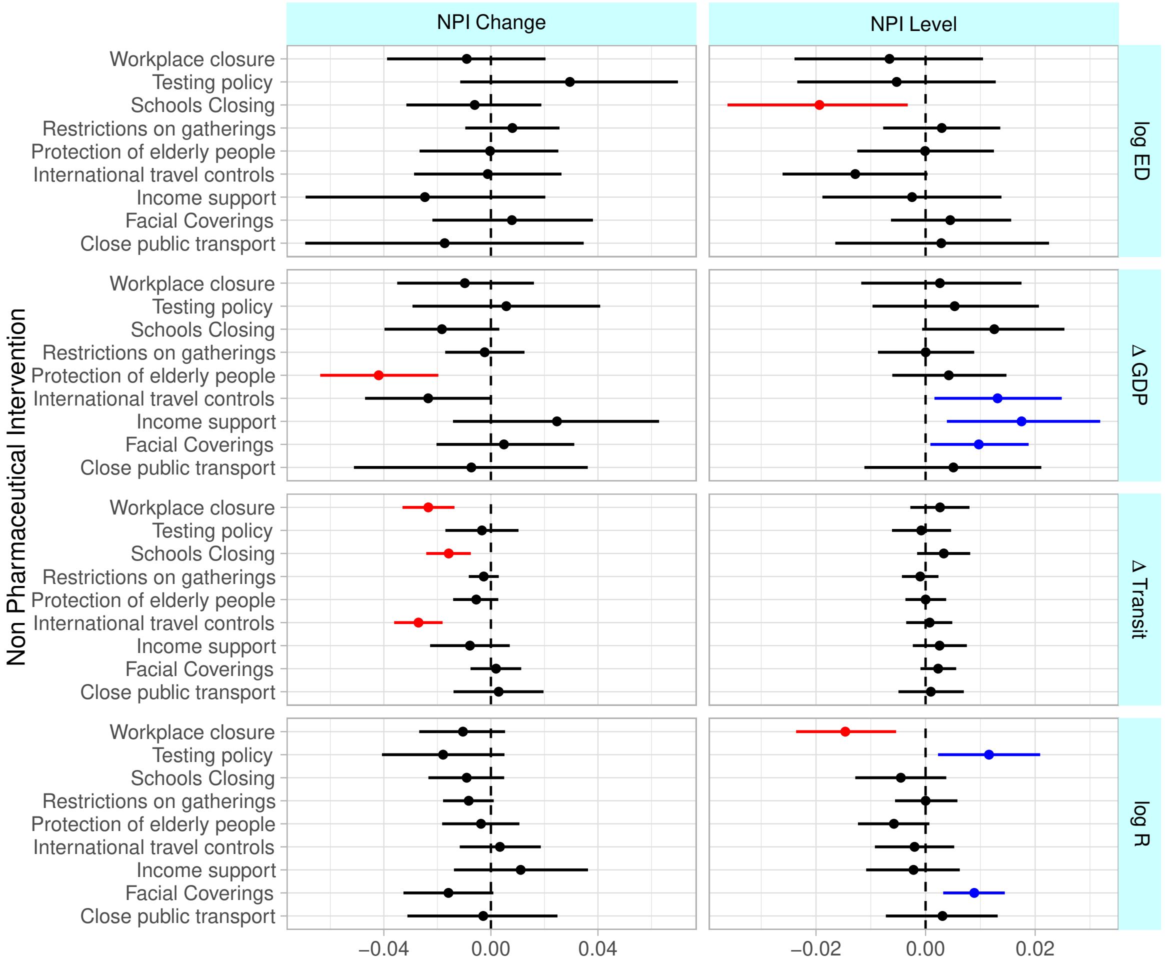


Figure C.15: NPI effect sizes for Model 3: Coefficient effect sizes (with 95% credible intervals) for each response variable. NPI changes (left column) and NPI Levels (right column) with NPI names listed on the vertical axis. Blue highlighted results indicate positive and significant coefficients and red indicates negative and significant coefficients.

## C.4 Country specific characteristics (Model 2)

| Measure | log ED | Delta GDP | Delta Transit | log R |
|---|---|---|---|---|
| Proportion households with member >60 | 0.651 | 0.271 | 0.240 | 0.217 |
| Poverty Rate, 2020 | 0.561 | 0.293 | 0.261 | 0.337 |
| Infanty Mortality Per 1000 Live Births | 0.550 | 0.179 | 0.278 | 0.373 |
| Number of Hospital beds per 100k population (2019, Eurostat) | 0.510 | 0.089 | 0.375 | 0.006 |
| GINI index (World Bank estimate), 2017 | 0.394 | 0.303 | 0.405 | 0.294 |
| Rural population (% of total population), 2017 | 0.371 | 0.111 | 0.142 | 0.167 |
| Labor Freedom (2018, Heritage Foundation) | 0.349 | 0.170 | 0.100 | 0.203 |
| Extent and Prevalence of Labour Informality | 0.285 | −0.036 | 0.016 | 0.172 |
| Average number of people in household, 2009-2018 | 0.188 | 0.171 | −0.044 | 0.064 |
| Prevalence of undernourishment (% of population, WB 2018) | 0.124 | 0.021 | 0.177 | 0.208 |
| Vulnerable Employment as % of total Employment (WB, 2018) | 0.097 | −0.203 | −0.117 | 0.339 |
| Net Forest Depletion % of GNI (WB, 2018) | −0.098 | 0.264 | 0.222 | 0.100 |
| % Face-to-Face sectors (2020, Eurostat) | −0.110 | −0.390 | −0.411 | 0.222 |
| Population in the largest city (% of urban population), 2018 | −0.207 | 0.084 | 0.045 | 0.233 |
| Individualism | −0.208 | −0.077 | −0.248 | −0.039 |
| Population density (people per sq. km of land area), 2018 | −0.241 | −0.302 | −0.294 | −0.181 |
| Ages 15-24 Employment to Population Ratio (WB, 2018) | −0.264 | −0.069 | −0.193 | 0.008 |
| Obedience | −0.287 | −0.042 | −0.365 | −0.054 |
| B40 share of income, 2020 | −0.290 | −0.254 | −0.248 | −0.215 |
| Proportion of Population Living Within 60 Minutes of an Urban Centre | −0.315 | −0.543 | −0.398 | −0.456 |
| Number of Physicians per 100k population (2019, Eurostat) | −0.319 | −0.216 | −0.148 | −0.213 |
| % of Seats Held by Women in National Parliament (WB, 2018) | −0.321 | −0.334 | −0.553 | −0.355 |
| Urban population (% of total population), 2018 | −0.371 | −0.111 | −0.142 | −0.167 |
| Ratio of Female to Male Labor Force Participation (WB, 2018) | −0.374 | −0.059 | −0.146 | −0.183 |
| Used the internet to pay bills in the past year (% age 15+), 2017 | −0.390 | −0.041 | −0.197 | −0.164 |
| Regulation Freedom (2018, Heritage Foundation) | −0.427 | −0.309 | −0.389 | −0.023 |
| Used the internet to pay bills or to buy something online in the past year (% age 15+), 2017 | −0.434 | −0.180 | −0.228 | −0.297 |
| Property Rights (2018, Heritage Foundation) | −0.484 | −0.249 | −0.354 | −0.237 |
| Electric power consumption (kWh per capita), 2014 | −0.485 | −0.082 | −0.184 | −0.334 |
| Health Expenditure as Fraction of GDP 2016 | −0.502 | −0.587 | −0.520 | −0.343 |
| Government Effectiveness estimates, 2018 | −0.559 | −0.219 | −0.276 | −0.287 |
| Proportion of Jobs That Are Teleworkable | −0.564 | −0.115 | −0.155 | −0.220 |
| Freedom House Global Freedom Score | −0.576 | −0.342 | −0.339 | −0.206 |
| Government Effectiveness percentile rank, 2018 | −0.583 | −0.248 | −0.273 | −0.293 |
| UN Human Development Index (WB, 2018) | −0.583 | −0.439 | −0.393 | −0.338 |
| 1st Principal Component of 6 Government Effectiveness Indices | −0.587 | −0.222 | −0.320 | −0.310 |
| Rule of Law (WB, 2018) | −0.592 | −0.285 | −0.413 | −0.343 |
| Individuals using the Internet (% of population) | −0.594 | −0.148 | −0.265 | −0.226 |
| RAND Infectious Disease Vulnerability Index | −0.600 | −0.354 | −0.491 | −0.426 |
| Population in urban agglomerations of more than 1 million (% of total population), 2018 | −0.606 | −0.357 | −0.422 | 0.038 |
| Control of Corruption Index (WB, 2018) | −0.612 | −0.261 | −0.331 | −0.336 |
| Financial institution account (% age 15+), 2017 | −0.658 | −0.389 | −0.378 | −0.330 |
| Life Expectancy at Birth (WB, 2018) | −0.766 | −0.664 | −0.587 | −0.495 |

Table C.10: Covariates: Correlation of country specific characteristics to country specific response variable intercepts across Government, Societal, economic and Healthcare characteristics.

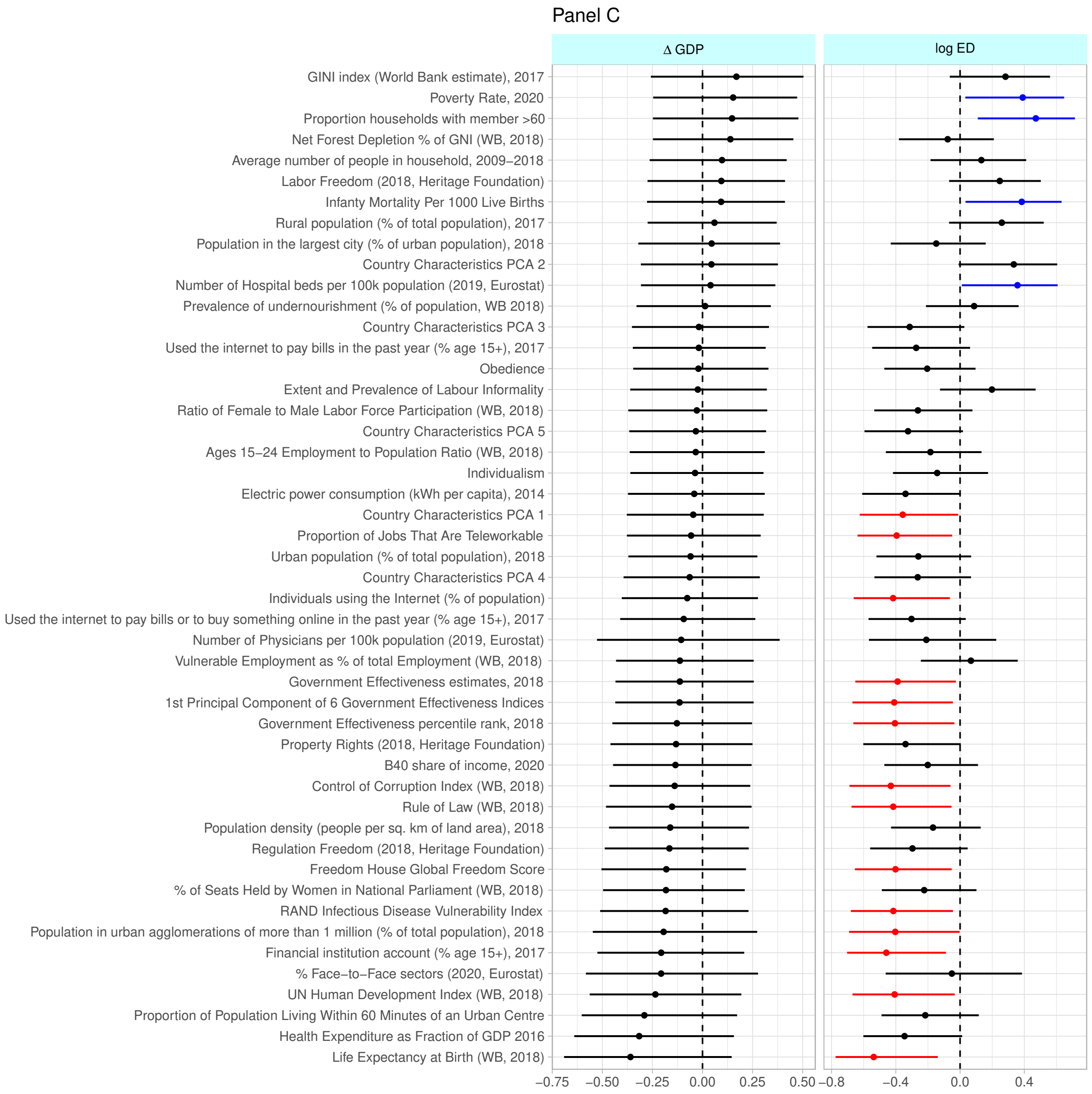


Figure C.16: Correlations of country specific characteristics with country specific intercepts for Δ GDP and log ED which are significant at the 90% CrI. Blue indicates positive and significant correlations and red indicates negative and significant correlations.

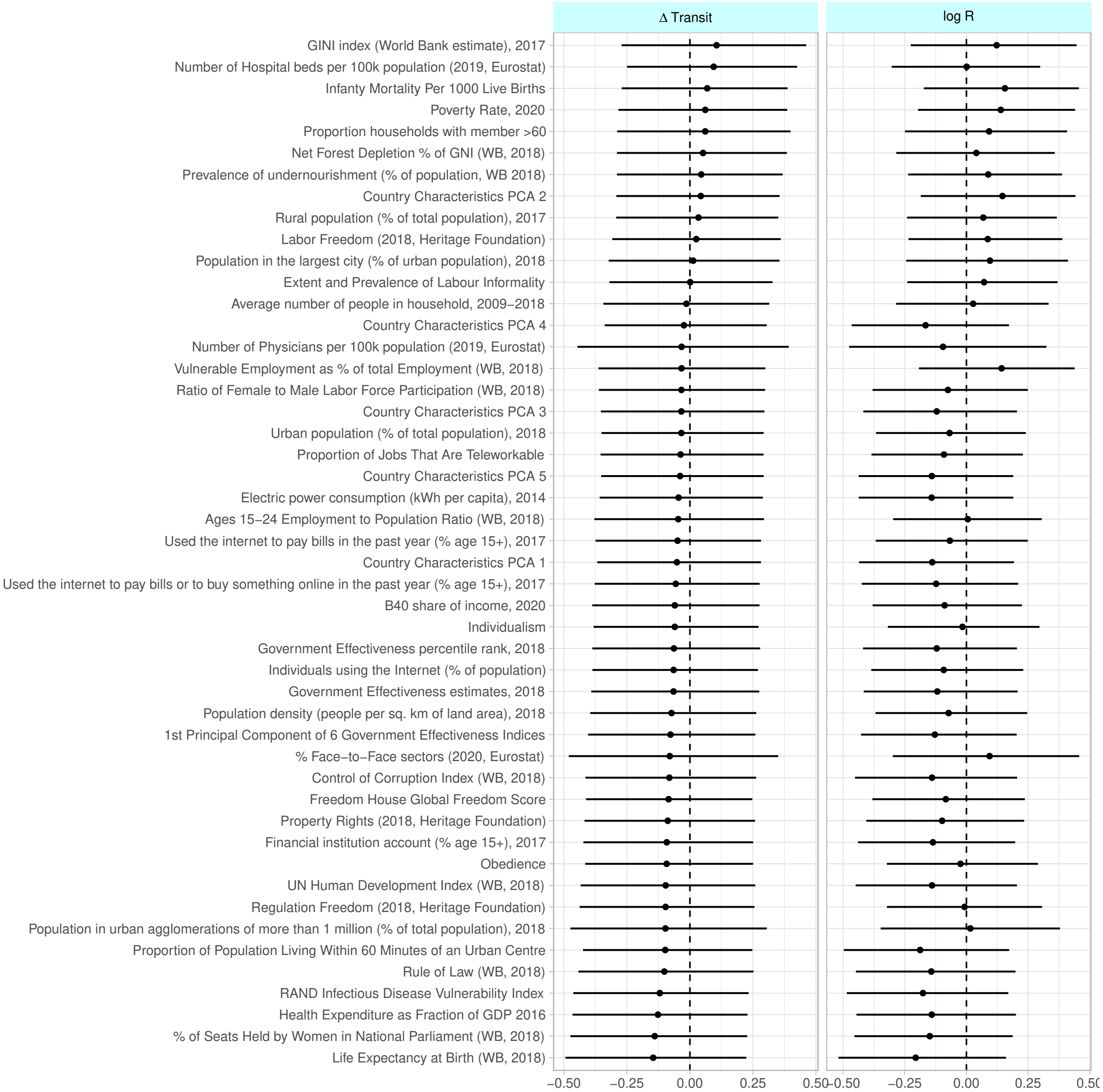


Figure C.17: Correlations of country specific characteristics with country specific intercepts for Δ Transit and log R (none significant at the 90% CrI).

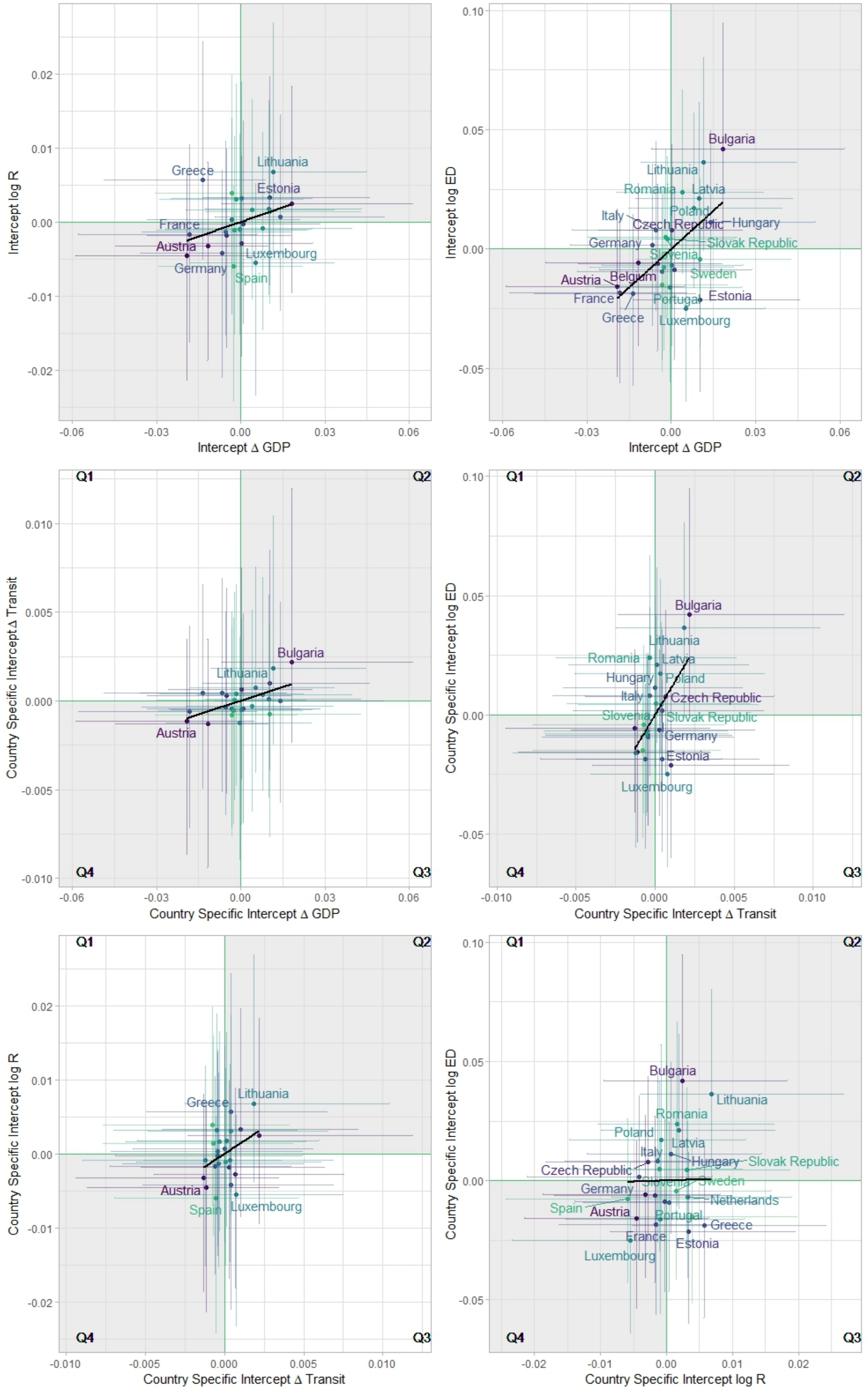


Figure C.18: Country Effects: Country Level Effects of Response Variables for all Response variable combinations. We highlight the 4 quadrants to display where countries are in the representation.

| Excluded Variable | ELPD-diff | SE-diff | CrI Lower Bound | CrI Upper Bound |
|---|---|---|---|---|
| Full Model | 0.000 | 0.000 | 0.000 | 0.000 |
| Δ Transit | -5.690 | 4.685 | -15.060 | 3.681 |
| Δ GDP | -9.984 | 5.896 | -21.777 | 1.808 |
| Δ GDP & Δ Transit | -14.264 | 7.387 | -29.039 | 0.511 |
| $logED$ | -40.741 | 10.350 | -61.440 | -20.042 |
| $logED$ & Δ Transit | -48.823 | 11.441 | -71.706 | -25.941 |
| Δ GDP & $logED$ | -51.353 | 12.469 | -76.291 | -26.415 |
| $logR$ | -69.044 | 13.484 | -96.012 | -42.076 |
| $logR$ & Δ Transit | -73.989 | 14.424 | -102.837 | -45.140 |
| Δ GDP & $logR$ | -84.924 | 14.819 | -114.563 | -55.286 |
| $logR$ & $logED$ | -117.163 | 18.069 | -153.301 | -81.026 |
| All Variables | -145.501 | 20.373 | -186.248 | -104.754 |

Table C.11: Leave-One-Out cross-validation (LOO-CV) to estimate the pointwise out of sample prediction accuracy of fitted Bayesian models. The full model is that described in the main text, excluded variables are removed from the prediction variables of the model (except for variables on themselves). Expected log pointwise predictive density (ELPD) differences and Standard Errors are reported.

## C.5 Forecast comparison

We are interested in the causal relationships of our response variables. Clearly, a cause cannot precede its the effect. If a variable $x$ affects another variable $y$, the former should help improve the predictions of the latter. We again use LOO-CV to estimate the pointwise out-of-sample prediction accuracy from our fitted model. We systematically remove predictive variables from the model: if variable x is removed, it is removed from all equations, except the equation of x on itself (e.g. removing $logR$ removes it from the right hand side of the equations of $logED$, Δ GDP and Δ Transit, but not the right hand side of $logR$ itself). We do this for each response variable individually, all combinations of two response variables and removing all response variables (in which case each variable uses only itself). The results are summarised in Table C.11, ordered by decreasing expected log pointwise predictive density (ELPD) difference.

The full model containing all variables has the best ELPD, although models excluding Δ Transit, Δ GDP or both of these are not statistically different at the 95% level from the full model. Excluding $logR$ or $logED$, and any combination containing these predictors, the difference is statistically significant. $logR$ is the most important predictive variable, as it leads to the largest negative ELPD differences, either on its own (compared to other single variable exclusions) or in combination with other variables excluded. Excluding all variables, so that each response variable is a function of only its past observations, leads to the largest ELPD negative difference indicating the worst performing model with respect to sample prediction accuracy. Together with the above vector-autoregressive coefficient estimates and the impulse response function, we have further evidence that transmission intensity increases excess deaths and negatively changes GDP.

To further assess the predictive performance of our model, we compare the 1-timestep ahead forecast from our model to the naive forecast, which assumes the next value of a response variable is the same as its current value, that is $\hat{x}(t+1) = x(t)$. We find that our model improves predictions of all response variables, with a reduction of root mean squared error of 8% for transmission intensity, 6% for excess deaths, 27% for Δ GDP and 32% for Δ Transit (Figure C.19). The improvements are due to improved forecasts in the tail of the distributions as opposed to the centre. This is expected, as the centre represents relatively stationary time periods. The model however does not have any awareness of e.g. holiday effects or other exogenous dynamics, and hence does not outperform the naive forecast during these periods. In the tails, i.e. during periods of high transmission intensity, the model performs better than the naive forecast.

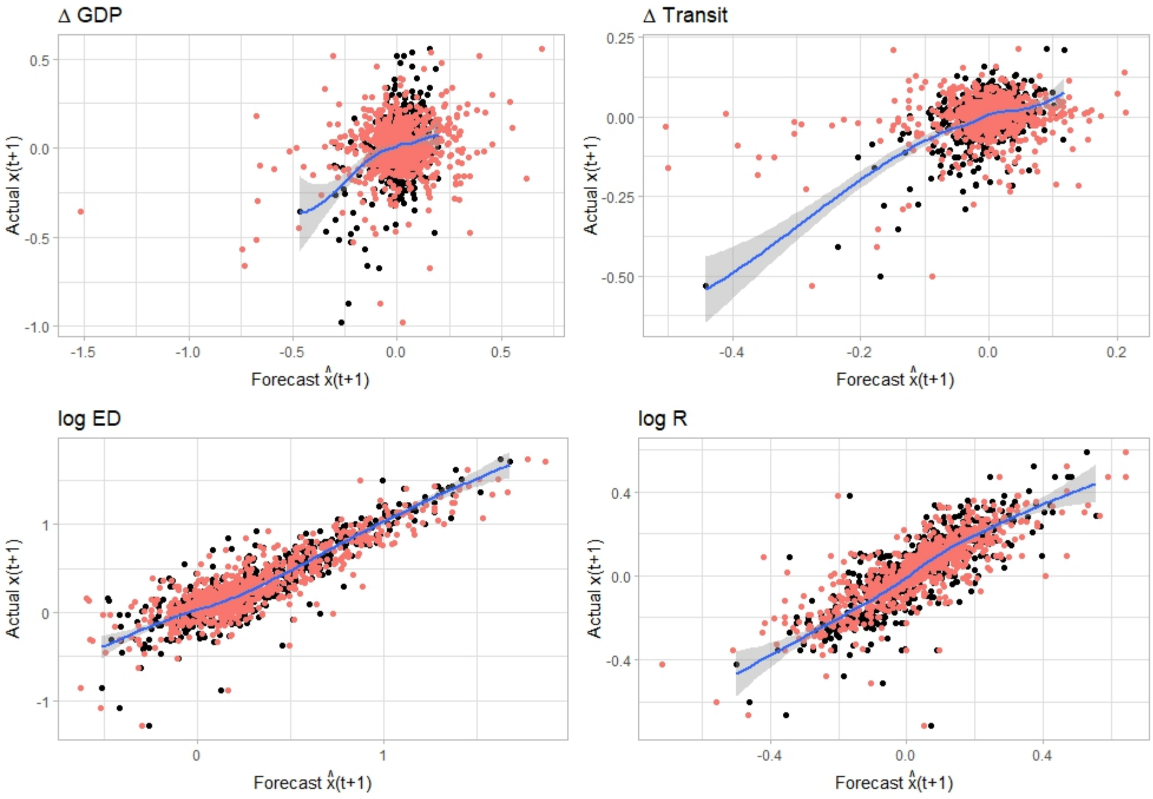


Figure C.19: Forecast Error: Plot of $\hat{x}(t+1)$ forecast vs actual $x(t+1)$. Black dots are forecast from model, Red dots are naive forecast $\hat{x}(t+1) = x(t)$

## C.6 Sensitivity Analysis

In order to check that no single country overly influenced the estimation, we re-estimated the model 25 times, each time leaving out one of the countries in the analysis. In Figure C.20, we plot the distribution of coefficients of the VAR component of the model. While there is some variation in the resulting coefficient estimates, this variation is generally small.

We also consider robustness for parameter choices in our model and estimation, such as $\tau$. The variable selection broadly follows the approach taken in [28].

There are a range of different specification for the model. Rather than estimating the model with no intercept we could introduce a global intercept and impose a constraint on the wild type variant coefficient to equal zero. The estimated coefficients are essentially unchanged by this.

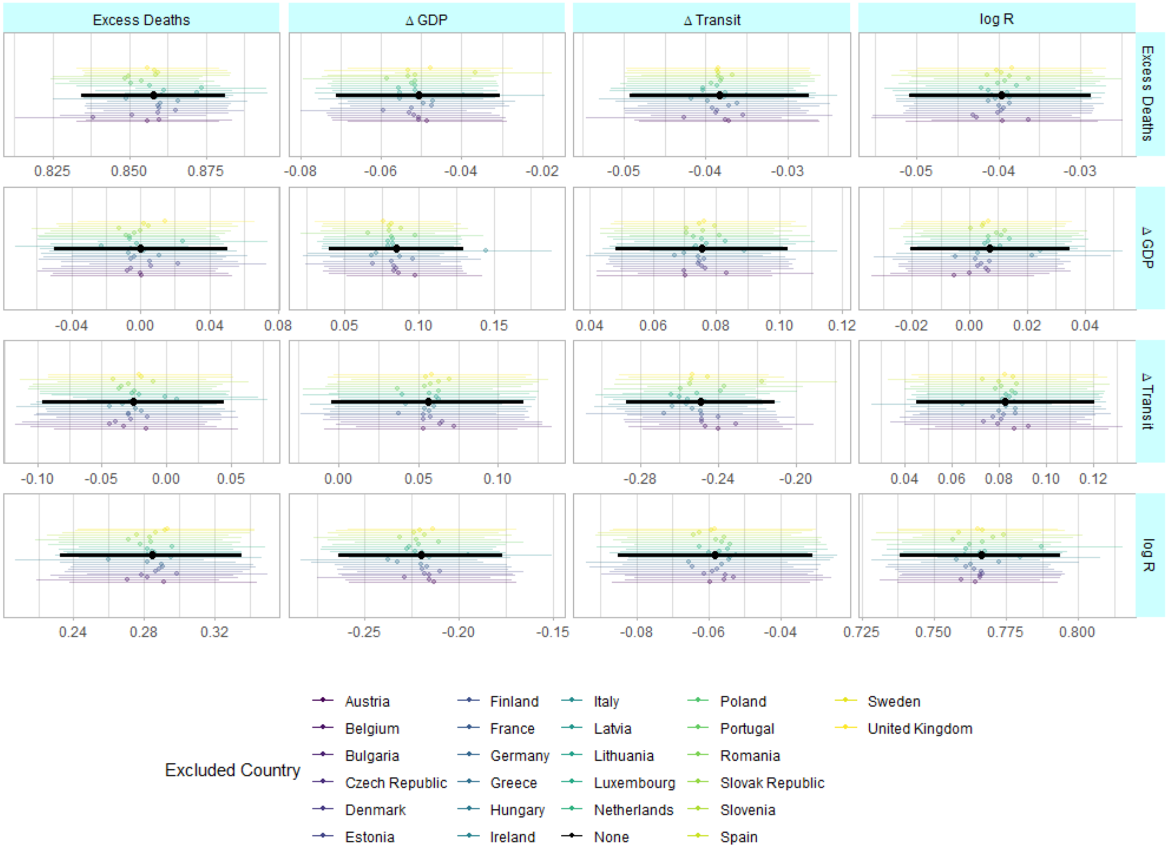


Figure C.20: Country Sensitivity Analysis: Marginal posterior density 90% confidence interval and mean for coefficients of the VAR component. Black line is the estimate for the full model, each colour represents the estimate of the model excluding that country.

## C.7 Convergence statistics

MCMC convergence statistics are available in Figure C.21 and show the rank-normalized split $\hat{R}$ score [30] and relative Effective Sample Size MCMC convergence statistics. Values of $\hat{R}$ close to 1 indicate convergence of the MCMC sampling algorithm. We note the sample properties are such $\hat{R} \leq 1.01$ for all estimated parameters [30] (Figure C.21). This implies that the posterior has converged and can be used for inference.

We used 2000 warmup samples and 2000 iterations per chain, for 4 chains. We also see that the relative effective sample size exceeds 0.5 for the majority of parameters, indicating low autocorrelation and fast mixing.

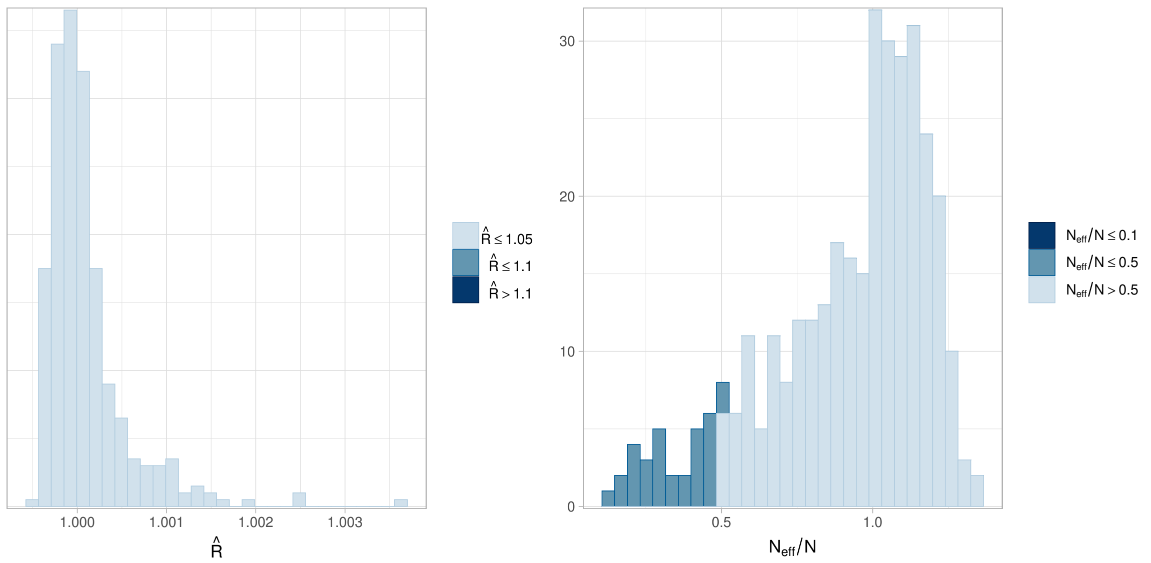


Figure C.21: MCMC convergence statistics (taken from a run using model values for all parameters as outlined in Table 1). Left: $\hat{R}$ values are close to 1, indicating convergence. Right: relative Effective sample size .

# References


1. Abbott S, Hellewell J, Thompson RN, Sherratt K, Gibbs HP, Bosse NI, Munday JD, Meakin S, Doughty EL, Chun JY, Chan YWD, Finger F, Campbell P, Endo A, Pearson CAB, Gimma A, Russell T, Flasche S, Kucharski AJ, Eggo RM, and Funk S. Estimating the time-varying reproduction number of SARS-CoV-2 using national and subnational case counts. Wellcome Open Research 2020 Dec 8; 5:112. DOI: 10.12688/wellcomeopenres.16006.2. Available from: http://dx.doi.org/10.12688/wellcomeopenres.16006.2
2. Arroyo-Marioli F, Bullano F, Kucinskas S, and Rondón-Moreno C. Tracking R of COVID-19: A new real-time estimation using the Kalman filter. PLOS ONE 2021 Jan 13; 16. Ed. by Sartorius B:e0244474. DOI: 10.1371/journal.pone.0244474. Available from: http://dx.doi.org/10.1371/journal.pone.0244474
3. Hale T, Petherick A, Phillips T, and Webster S. Variation in government responses to COVID-19. BSG Working Paper Series 2020; BSG-WP-2020/031. Available from: https://www.bsg.ox.ac.uk/sites/default/files/2020-04/BSG-WP-2020-031-v4.0_0.pdf
4. Mathieu E, Ritchie H, Ortiz-Ospina E, Roser M, Hasell J, Appel C, Giattino C, and Rodés-Guirao L. A global database of COVID-19 vaccinations. Nature human behaviour 2021; 5:947–53
5. EuroStat. eurostat - Your Key to European Statistics. 2022. Available from: https://ec.europa.eu/eurostat/data/database
6. Hodcroft, Emma B. CoVariants: SARS-CoV-2 Mutations and Variants of Interest. 2021. Available from: https://covariants.org/

7. Hanushek EA and Woessmann L. The economic impacts of learning losses. 2020. DOI: https://doi.org/https://doi.org/10.1787/21908d74-en. Available from: https://www.oecd-ilibrary.org/content/paper/21908d74-en
8. Mathieu E, Ritchie H, Rodés-Guirao L, Appel C, Giattino C, Hasell J, Macdonald B, Dattani S, Beltekian D, Ortiz-Ospina E, and Roser M. Coronavirus Pandemic (COVID-19). Our World in Data 2020. https://ourworldindata.org/coronavirus
9. The Economist and Solstad S. The pandemic's true death toll. 2021. Available from: https://www.economist.com/graphic-detail/coronavirus-excess-deaths-estimates
10. Google LLC. Google COVID-19 Community Mobility Reports. 2022. Available from: https://www.google.com/covid19/mobility/
11. World Bank. World Bank Open Data - Free and open access to global development data. 2022. Available from: https://data.worldbank.org/
12. Lakner C, Mahler D, Negre M, and Prydz E. How Much Does Reducing Inequality Matter for Global Poverty? Global Poverty Monitoring Technical Note, World Bank, Bank, W., Washington, DC., 32 pp. 2020
13. Dingel JI and Neiman B. How many jobs can be done at home? Journal of public economics 2020; 189:104235
14. IMF. IMF Data Portal. 2022. Available from: https://data.imf.org/?sk=388dfa60-1d26-4ade-b505-a05a558d9a42
15. FED. Federal Reserve Economic Data. 2022. Available from: https://fred.stlouisfed.org/
16. OECD. Catalogue of OECD databases. Data across 38 member countries. 2022. Available from: https://data.oecd.org/searchresults/?r=+f/type/datasets
17. Chen S, Igan D, Pierri N, and Presbitero A. Tracking the Economic Impact of COVID-19 and Mitigation Policies in Europe and the United States. IMF Working Paper. 2020 Jul
18. Cerdeiro D, Komaromi A, Liu Y, and Saeed M. World Seaborne Trade in Real Time: A Proof of Concept for Building AIS-based Nowcasts from Scratch. IMF Working Paper. 2020 May 14. Available from: https://www.imf.org/en/Publications/WP/Issues/2020/05/14/World-Seaborne-Trade-in-Real-Time-A-Proof-of-Concept-for-Building-AIS-based-Nowcasts-from-49393
19. Deb P, Furceri D, Ostry JD, and Tawk N. The Economic Effects of COVID-19 Containment Measures. Open Economies Review 2021 Dec 14; 33:1–32. DOI: 10.1007/s11079-021-09638-2. Available from: http://dx.doi.org/10.1007/s11079-021-09638-2
20. Ashraf BN. Economic impact of government interventions during the COVID-19 pandemic: International evidence from financial markets. Journal of Behavioral and Experimental Finance 2020 Sep; 27:100371. DOI: 10.1016/j.jbef.2020.100371. Available from: http://dx.doi.org/10.1016/j.jbef.2020.100371
21. Heritage Foundation. Heritage Foundation - Index of Economic Freedom. 2022. Available from: https://www.heritage.org/index/explore
22. Freedom House. Freedom House Global Freedom Score. 2022. Available from: https://freedomhouse.org/countries/freedom-world/scores
23. Moore M, Gelfeld B, Okunogbe AT, and Paul C. Identifying Future Disease Hot Spots: Infectious Disease Vulnerability Index. Santa Monica, CA: RAND Corporation, 2016. DOI: 10.7249/RR1605
24. Imperial College London/YouGov. COVID-19 behaviour tracker. 2020. Available from: https://www.imperial.ac.uk/global-health-innovation/what-we-do/our-response-to-covid-19/covid-19-behaviour-tracker/
25. Pollock III PH and Edwards BC. The essentials of political analysis. Cq Press, 2019
26. United Nations. undata - A world of information. 2022. Available from: https://data.un.org
27. Schulz JF, Bahrami-Rad D, Beauchamp JP, and Henrich J. The Church, intensive kinship, and global psychological variation. Science 2019; 366:eaau5141
28. Liu Y, Morgenstern C, Kelly J, Lowe R, and Jit M. The impact of non-pharmaceutical interventions on SARS-CoV-2 transmission across 130 countries and territories. BMC Medicine 2021 Feb 5; 19. DOI: 10.1186/s12916-020-01872-8. Available from: http://dx.doi.org/10.1186/s12916-020-01872-8
29. Turner D, Égert B, Guillemette Y, and Botev J. The tortoise and the hare: The race between vaccine rollout and new COVID variants. 2021. DOI: https://doi.org/https://doi.org/

10.1787/4098409d-en. Available from: https://www.oecd-ilibrary.org/content/paper/4098409d-en
30. Vehtari A, Gelman A, Simpson D, Carpenter B, and Bürkner PC. Rank-normalization, folding, and localization: An improved Rˆ for assessing convergence of MCMC (with discussion). en. Bayesian Anal. 2021 Jun; 16
